# Bacterial Inoculant and Sucrose Amendments Improve the Growth of *Rheum palmatum* L. by Reprograming Its Metabolite Composition and Altering Its Soil Microbial Community

**DOI:** 10.3390/ijms23031694

**Published:** 2022-02-01

**Authors:** Yuan Tian, Yang Liu, Liang Yue, Constantine Uwaremwe, Xia Zhao, Qin Zhou, Yun Wang, Ruoyu Wang

**Affiliations:** 1 Gansu Gaolan Field Scientific Observation and Research Station for Agricultural Ecosystem, Northwest Institute of Eco-Environment and Resources, Chinese Academy of Sciences, Lanzhou 730000, China; ty19920101@163.com (Y.T.); yangliu1229@126.com (Y.L.); 15002517498@163.com (L.Y.); ricikuku@yahoo.fr (C.U.); zhaoxia@lzb.ac.cn (X.Z.); zhouqin022@lzb.ac.cn (Q.Z.); hp.wangyu@gmail.com (Y.W.); 2 College of Resources and Environment, University of Chinese Academy of Sciences, Beijing 100049, China; 3 Key Laboratory of Desert and Desertification, Northwest Institute of Eco-Environment and Resources, Chinese Academy of Sciences, Lanzhou 730000, China

**Keywords:** biocontrol, carbon sources, herbs, metabolome, nutrient utilization, plant–microbe interaction

## Abstract

*Rheum palmatum* L. is an important traditional Chinese medicinal herb now in demand worldwide. Recently, the theoretical framework suggested that sucrose triggers colonization of PGPM (plant growth-promoting microbes) in the rhizosphere, but their interactions on the plant remain largely unknown. Here, we applied three concentrations of both *Bacillus amyloliquefaciens* EZ99 inoculant (1.0 × 10^5^, 1.0 × 10^6^, and 1.0 × 10^7^ colony-forming units (CFU)/mL, denoted as LB, MB, and HB, respectively) and sucrose (0.15, 1.5, and 15 g/L, denoted as LS, MS, and HS, respectively) to investigate their co-effects on *R. palmatum* in a field experiment. The results showed that LB + MS (1.0 × 10^5^ CFU/mL *Bacillus* + 1.5 g/L sucrose) and LB + LS (1.0 × 10^5^ CFU/mL *Bacillus* + 0.15 g/L sucrose) treatments significantly increased root fresh weight (*p* ≤ 0.05). Metabolite analysis revealed that the treatment LB + LS significantly increased the relative content of major active components in rhubarb, namely anthraquinones and phenolic compounds, by 1.5% and 2.3%. Although high sucrose addition increased the activities of certain soil enzymes, the LB + LS treatment significantly increased total potassium (TK), whereas it decreased available potassium (AK), which facilitated the potassium utilization in rhizosphere soil. Furthermore, rhizosphere microbiomes revealed that fungal diversity was augmented in LB + LS treatment, in which the common causative fungal pathogen *Fusarium* spp. showed an effective suppression. Additionally, the redundancy analysis and Spearman correlations revealed a positive relationship of *Sphingomonas* associated with change in potassium bioavailability. Altogether, our findings suggest that the combined application of a bacterial inoculant and sucrose can improve the growth and quality of *R. palmatum*, and stimulate uptake of plant nutrients that contribute to alter the microbial community for biocontrol potential. Hence, this work not only has broad application prospects across economical plants, but also emphasizes agroecological practices for sustainable agriculture.

## 1. Introduction

The sustainable promotion of plant growth and productivity requires improving soil quality and reducing the disease incidence in an ecologically friendly way [1]. Compared with the excessive use of chemical fertilizers which cause environmental hazards, degrade soil properties, and consequently harm crops and human health, using biofertilizers could be an environmentally sound option since their effects are sustainable. Biofertilizers are applied to the soil or plant to stimulate its growth via the production of phytohormones, resistance to pathogens, improved soil nutrients uptake, and resilient microbial structure [2].

Forming the core of biofertilizers are PGPMs (plant growth-promoting microbes), that colonize plant roots in the rhizosphere and induce plant growth through either direct or indirect mechanisms [3,4]. The rhizosphere is a “hot spot” that is defined as the narrow zone of soil (<2 mm) associated with plant roots [5,6,7]. There are many bacterial and fungal species that can function as PGPM, of which *Bacillus* members are well-described in the literature for successfully promoting plant growth in diverse ways. *Bacillus* isolates colonize host plant roots and promote plant growth by producing bio compounds, such as the hormone indole-3-acetic acid (IAA), as well as spermidine and 2,3-butanediol [4,8,9], by defending against pests and pathogens by producing antibiotic substances such as hydrogen cyanide (HCN), chitinase, and siderophores [10,11,12], and by competing for nutrients and micro-niches, as well as induction of plant resistance [13,14,15]. Some studies have reported on the synergistic and antagonistic effects of PGPM, as well as other microorganisms within the rhizosphere and beyond (in bulk soil), which could indirectly facilitate plant growth [16,17,18]. Moreover, *Bacillus*-based biofertilizers display high resilience to heat and drought due to their endospores-forming ability. Hence, products containing *Bacillus* spp. have been used as seed dressings or a biocontrol method in numerous crops and some herbs [19,20,21].

Recently, the notion that applying biofertilizer individually is limiting and that rather its combination with other amendments required for efficacy is gaining traction [22]. Inorganic amendments, such as nitrogen [23,24], phosphorus [25,26,27], and potassium [27,28,29], can boost nutrient availability in vegetables and crops. In general, soil is usually a carbon-limited state, and less than 5% of total bacteria are in an active state under such conditions [30]. Carbon sources, especially the most common sucrose, could impact bacteria as a direct energy source for growth, as well as forming their chemotaxis gradient [31,32]. To our knowledge, the PGPM arbuscular mycorrhizal fungi (AMF), coupled with fructose addition to the hyphosphere of maize plants, have increased soil phosphatase activity and resulted in rhizosphere priming effects [33]. However, there are no field investigations on the effect of sucrose, coupled with any *Bacillus* spp. for plant growth. To elucidate how diverse PGPM respond to the small molecular carbon addition such as sucrose and their co-effects on the rhizosphere microecology is a question of utmost importance.

*Rheum palmatum* L. (Polygonaceae) is a traditional Chinese medicinal plant. Rhubarb, called ‘Dahuang’ in Chinese, is the dry root and root stock used as a medicinal herb of *Rheum* species, namely *R. palmatum* L. (http://mpns.kew.org) (accessed on 20 January 2022), *R. tanguticum* (Maxim. Ex Regel) Balf. (http://mpns.kew.org) (accessed on 20 January 2022), and *R. officinale* Baill. (http://mpns.kew.org) (accessed on 20 January 2022). *Rheum* has been used for thousands of years in China, and is officially listed in the Pharmacopoeia of China, Japan, and Europe [34,35]. According to many studies [36,37,38], more than 100 secondary metabolites exist in *Rheum* spp. [39], of which the major bioactive components are anthraquinones, a group of phenolic compounds whose basic structure consists of anthraquinone rings and the substituents [40], including rhein, emodin, aloe-emodin, chrysophanol, and physcion. Thus, *R. palmatum* possesses various therapeutic functions, such as purgative, clearing heat, detoxification, breaking blood stasis, diabetic control, and relieving jaundice [41,42,43]. *R. palmatum* is a high-altitude perennial herb that is sensitive to high temperature, with its wild populations mainly distributed in Southwest and Northwest China, such as Gansu, Sichuan, and Qinghai provinces [44]. Due to the limited distribution, overexploitation, and diseases, the wild sources of *R. palmatum* are now endemic to China. Despite artificial cultivation having been developed to ensure the yield of *Rheum*, the frequent pest outbreaks and incidence of various diseases, together with the lower content of effective medical ingredients, are major issues that threaten its long-term production. Hence, it is imperative to establish sustainable management practices (fertilizer, biocontrol of pathogens) to improve the yield and quality of *R. palmatum* as an efficacious medicine.

Soil is the natural habitat for microorganisms, where they play a significant role in soil processes and the determination of plant productivity [45]. Due to the high potential of producing secondary metabolites and the comprehensive communication within the microbial community, the interaction of an inoculant and the indigenous microbial community needs to be extensively analyzed and reported [21,46,47]. Although application of the commercially available strain *B. amyloliquefaciens* FZB42 may have a negative impact on the indigenous microbial rhizosphere community [46], our recent work [21] suggested its biocontrol efficiency on root rot disease of *Angelica sinensis* caused by *Fusarium* and other causative pathogens, such as *Gibberella*, *Cylindrocarpon*, and *Plectosphaerella*. Thus, the need to understand the plant microecology in a microecosystem such as the rhizosphere is an essential first step to obtain better results in the field when inoculating PGPM.

To attain profitable productivity, *R. palmatum* requires highly fertilized soils [48], yet surprisingly, there is a dearth of information on the effects of microbial inoculants on *R. palmatum*, much less for carbon amendment. In this study, to fill the knowledge gap of how PGPM with sucrose amendment impacts on plants, we first applied three levels of *B. amyloliquefaciens* EZ99 inoculants and sucrose to determine their co-effects on *R. palmatum* in a field experiment, as characterized by metabolome and rRNA-gene-based sequencing.

## 2. Results

### 2.1. The Effects of PGPM and Sucrose Applications on R. palmatum Growth and Yield

**Figure 1:**
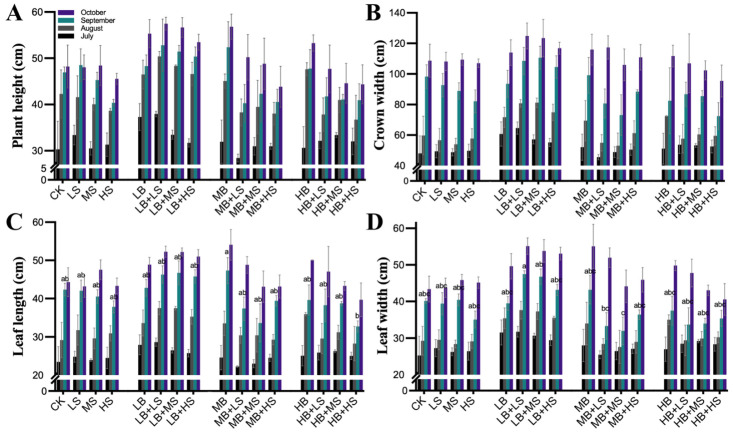
Influence of PGPM (plant growth-promoting microbes) and sucrose applications on *R. palmatum* growth parameters, of plant height (**A**), crown width (**B**), leaf length (**C**), and leaf width (**D**) over time. CK, water control. LS (low sucrose), MS (medium sucrose), and HS (high sucrose) denote 0.15, 1.5, and 15 g/L of sucrose, respectively. LB (low *Bacillus*), MB (medium *Bacillus*), and HB (high *Bacillus*) denote 1.0 × 10^5^, 1.0 × 10^6^, and 1.0 × 10^7^ CFU/mL of *Bacillus* inoculant, respectively. Values are the mean ± SE (*n* = 30). The comparison was performed within a given month, for which different lowercase letters indicate a significant difference at *p* ≤ 0.05. Absence of lowercase letters indicates that differences were non-significant.

Under the field condition, the growth parameters of *R. palmatum* at various growth times (July, August, September, and October) are shown in Figure 1. Compared with control groups (CK), the plant height, crown width, leaf length, and leaf width treated with any three concentrations of the bacterial inoculant (LB, MB, and HB) were increased. Our results showed two clear inverted V-shaped trends, with an outer peak appearing in the four LB-containing treatments (LB, LB + LS, LB + MS, and LB + HS). Although the HB treatment increased the growth of *R. palmatum*, it decreased the growth when coupled with sucrose, especially under the HB + HS treatment. This indicates that a high level of bacterial inoculant (1.0 × 10^7^ CFU/mL) amended with a high level of sucrose (15 g/L) suppressed the plant growth-promoting function of PGPM.

**Figure 2:**
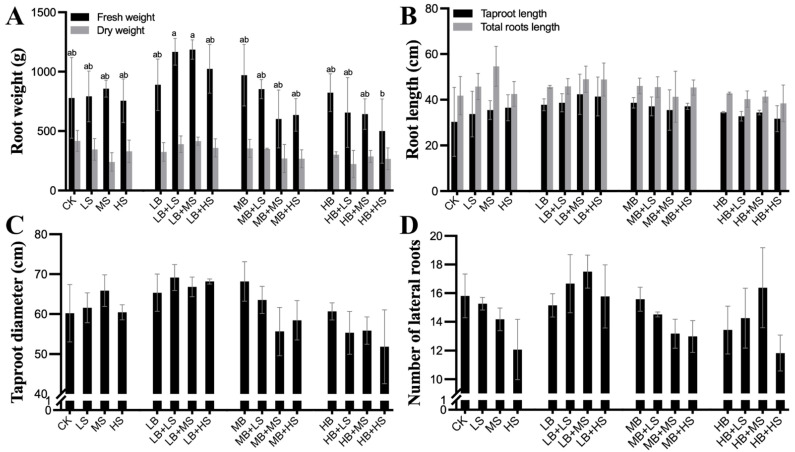
Influence of PGPM (plant growth-promoting microbes) and sucrose on *R. palmatum* roots during the harvesting stage. Root parameters in November are shown: root weight (**A**), root length (**B**), taproot diameter (**C**), and the number of lateral roots (**D**). Values are the mean ± SE (*n* = 30). CK, water control. LS (low sucrose), MS (medium sucrose), and HS (high sucrose) denote 0.15, 1.5, and 15 g/L of sucrose, respectively. LB (low *Bacillus*), MB (medium *Bacillus*), and HB (high *Bacillus*) denote 1.0 × 10^5^, 1.0 × 10^6^, and 1.0 × 10^7^ CFU/mL of *Bacillus* inoculant, respectively. The comparison was performed within root parameters, with different lowercase letters indicating a significant difference at *p* ≤ 0.05. Absence of lowercase letters indicates that differences were non-significant.

At harvest time, the changes in plot yield were also determined (Figure 2). Consistent with the growth results in two clear inverted V-shaped trends, the fresh weight of *R. palmatum* roots under the LB + LS and LB + MS treatments were the most significantly improved (Figure 2A). We further analyzed the changes in taproot and lateral roots (Figure 2B–D), and found that the greatest root length and number of lateral roots were obtained in LB + MS, whereas the highest taproot diameter was obtained under LB + LS, which implies that the LB + MS treatment stimulated the number and length of lateral roots, while LB + LS promoted taproot thickness to improve the root weight of *R. palmatum*.

### 2.2. Metabolic Differences of R. palmatum Roots under the PGPM and Sucrose Treatments

**Figure 3:**
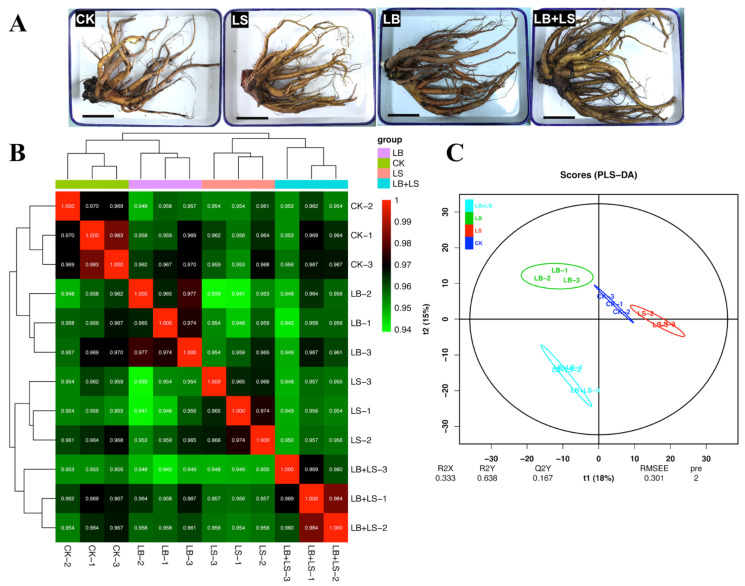
The evaluations of rhubarb (*R. palmatum*) widely targeted metabolomics (*n* = 3). (**A**) Appearance of rhubarb under the PGPM (plant growth-promoting microbes) and sucrose treatments. Scale bar = 10 cm. (**B**) Correlation heatmap and (**C**) partial least squares discriminant analysis (PLS-DA) of metabolic profiles in rhubarb (*R. palmatum*). The clustering Spearman rank correlations and the PLS-DA analysis were performed on the platform BMKCloud (www.biocloud.net) (accessed on 20 January 2022). The color gradient of corresponding correlation values represents the strength of the correlation between two variables. CK, water control; LS (low sucrose), 0.15 g/L of sucrose; LB (low *Bacillus*), 1.0 × 10^5^ CFU/mL of *Bacillus*; LB + LS, 1.0 × 10^5^ CFU/mL of *Bacillus* + 0.15 g/L of sucrose.

We next focused on the LB (1.0 × 10^5^ CFU/mL of *Bacillus*) combined with LS (0.15 g/L of sucrose) to explore potential mechanism(s) underpinning their effects, through the lens of widely targeted metabolomics (Figure 3A). A total of 12 fresh rhubarb root samples were characterized and 800 annotated metabolites were identified, mainly consisting of 38 carboxylic acids and derivatives, 15 organooxygen compounds, 14 fatty acyls, and 11 organonitrogen compounds (Supplementary Table S1). The global-targeted metabolome profiling approach with GC-TOF-MS was subjected to correlation partial least squares discriminant analysis (PLS-DA). As evinced by the heatmap (Figure 3B), the Spearman correlation coefficients among three biological pairings all exceeded 0.94, which emphasized the high reproducibility of the rhubarb metabolome. The PLS-DA score plot was then derived to assess the differences among these rhubarb samples (Figure 3C), and this revealed a clear separation across the treatments. Furthermore, the supervised orthogonal projections to latent structures discriminant analysis (OPLS-DA) modeling also uncovered significant differentiation in these rhubarb comparisons and the model was stable and reliable (Supplementary Figure S1). Briefly, the results indicated a robust quality of rhubarb’s metabolome profiling.

**Figure 4:**
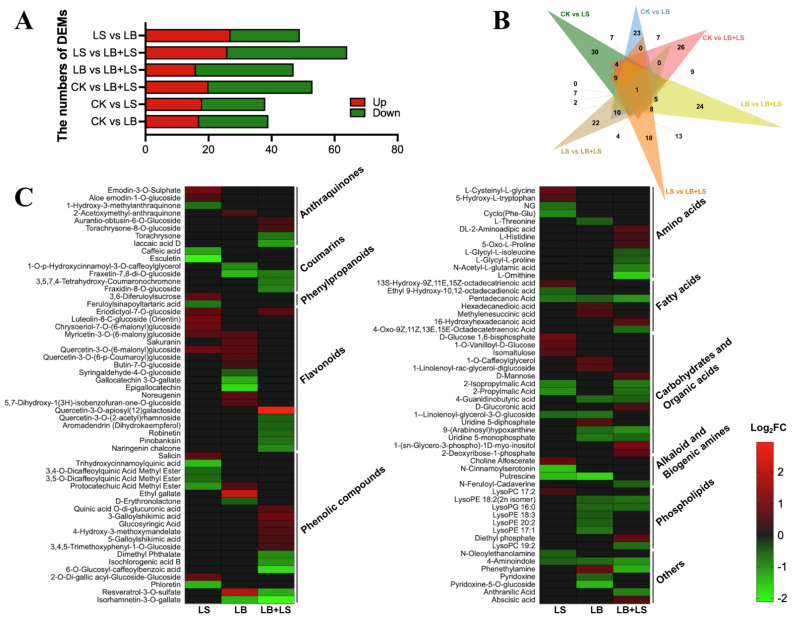
Widely targeted metabolomic analysis of *R. palmatum* under PGPM (plant growth-promoting microbes) and sucrose treatments (*n* = 3). (**A**) Number of differentially expressed metabolites (DEMs) among each comparison. (**B**) Venn diagram depicting number and overlapping DEMs from the metabolic profiles. (**C**) Heatmap analysis of DEMs among the samples. CK, water control; LS (low sucrose), 0.15 g/L of sucrose; LB (low *Bacillus*), 1.0 × 10^5^ CFU/mL of *Bacillus*; LB + LS, 1.0 × 10^5^ CFU/mL of *Bacillus* + 0.15 g/L of sucrose.

Using the screening criteria of the variable importance in projection (VIP) ≥ 1, fold change ≥ 1.5, and *p*-value ≤ 0.05, there were 38, 39, 53, 47, 64, and 49 differentially expressed metabolites (DEMs) significantly identified in CK vs. LS, CK vs. LB, CK vs. LB + LS, LB vs. LB + LS, LS vs. LB + LS, and LS vs. LB, respectively (Figure 4A; Supplementary Table S2). The most encountered DEMs were in the LS vs. LB + LS pair, with 26 upregulated and 38 downregulated. Moreover, the numbers of up/downregulated metabolites in each comparison were also visualized in a volcano plot (Supplementary Figure S2). The corresponding Venn diagram illustrates the unique and shared DEMs in the pairs across all comparisons (Figure 4B): evidently just one DEM (N-cinnamoyl serotonin) was shared by all six comparison pairs, implying that this metabolite is essential. Specifically, 30, 23, 26, 24, 18, and 22 DEMs were respectively uniquely found in CK vs. LS, CK vs. LB, CK vs. LB + LS, LB vs. LB + LS, LS vs. LB + LS, and LS vs. LB (Supplementary Table S2).

To uncover the full range of metabolites associated with the sucrose and PGPM applications, we further compared the DEMs of LS, LB, and LB + LS treatments with CK in a heatmap. As Figure 4C shows, eight kinds of anthraquinones, the major constituents of rhubarb, were differentially identified in the three comparisons. Relative to CK, the LS rhubarbs accumulated the highest level of emodin-3-O-sulphate and aloe emodin-1-O-glucoside, but had diminished levels of 1-hydroxy-3-methylanthraquinone. The LB + LS group accumulated the highest level of aurantio-obtusin-6-O-glucoside and torachrysone-8-O-glucoside, but reduced levels of torachrysone and laccaic acid D, while the rhubarbs under the LB treatment accumulated only 2-acetoxymethyl-anthraquinone. For the six coumarins, they all showed a downregulated level under the treatments. In contrast, the phenylpropanoids only varied in LS-treated rhubarb, with 3,6-diferuloylsucrose undergoing upregulation and feruloylsinapoyltartaric acid incurring downregulation. Numerous flavonoids were identified (18), including flavones and flavonols, with these being highly expressed in rhubarbs under either the LS or LB treatment, but with a downregulated level in LB + LS. Another large group was that of phenolic compounds (19), whose accumulation reached high levels in the LB + LS rhubarbs. Notably, the resveratrol-3-O-sulfate, a stilbene, was highly accumulated in rhubarbs under the LB treatment. Furthermore, for carbohydrates and organic acids, a global upregulation of saccharides was observed under the treatments of LS and LB, whereas a downregulation of organic acids was evident under LB + LS treatment.

We then conducted a metabolic enrichment analysis of DEMs in the Kyoto Encyclopedia of Genes and Genomes (KEGG) pathways (Supplementary Figure S3). These results revealed ether lipid metabolism (ko00565), starch and sucrose metabolism (ko00500), and glycolysis/gluconeogenesis (ko00010) as the significantly enriched pathways in the LS-treated rhubarbs, pyrimidine metabolism (ko00240), arginine and proline metabolism (ko00330), and C5-branched dibasic acid metabolism (ko00660) as the significantly enriched pathways in the LB-treated rhubarbs, and biosynthesis of amino acids (ko01230), 2-oxocarboxylic acid metabolism (ko01210), and arginine biosynthesis (ko00220) as the significantly enriched pathways in the LB + LS-treated rhubarbs (Supplementary Table S3).

### 2.3. Soil Properties, Enzyme Activities, and Microbial Biomass under the PGPM and Sucrose Treatments

**Figure 5:**
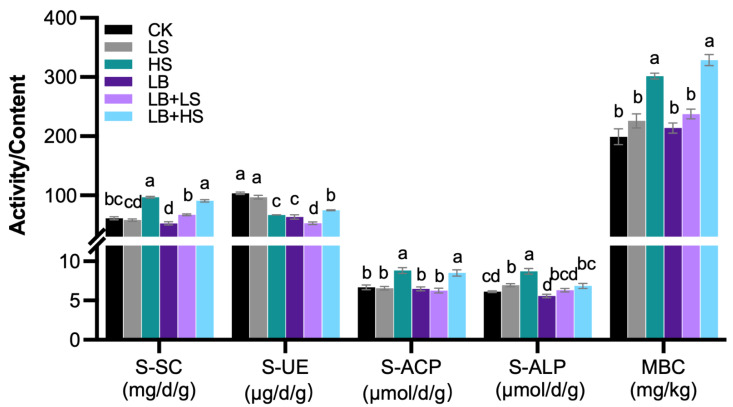
Soil enzyme activities (*n* = 3) and microbial biomass carbon (*n* = 6) in response to the PGPM (plant growth-promoting microbes) and sucrose treatments. S-SC, sucrase; S-UE, urease; S-ACP, acid phosphatase; S-AKP, alkaline phosphatase; MBC, microbial biomass carbon, CK, water control; LS (low sucrose), 0.15 g/L of sucrose; HS (high sucrose), 1.5 g/L of sucrose; LB (low *Bacillus*), 1.0 × 10^5^ CFU/mL of *Bacillus*; LB + LS, 1.0 × 10^5^ CFU/mL of *Bacillus* + 0.15 g/L of sucrose; LB + HS, 1.0 × 10^5^ CFU/mL of *Bacillus* + 1.5 g/L of sucrose. Values are the mean ± SE. Different lowercase letters indicate a significant difference at *p* ≤ 0.05.

The physicochemical properties of rhizosphere and bulk soils at the end of the experiment were presented in Table 1. Generally, the soil pH, soil water content (SWC), soil organic matter (SOM), total carbon (TC), total nitrogen (TN), and carbon to nitrogen ratio (C/N) ratio were fairly stable, both in rhizosphere and bulk soils, and likewise in terms of ammonium nitrogen (NH_4_-N). Intriguingly, however, the nitrate nitrogen (NO_3_-N) in bulk soil was exceptionally high (33.19 ± 2.98 mg/kg) when compared to the rhizosphere soil, which implies that the microorganisms in the rhizosphere possessed a high utilization rate of nitrogen. Furthermore, the amount of available phosphorus and total phosphorus in HS rhizosphere soils were significantly increased, which indicated that the high sucrose addition without any PGPM may have provided the carbon needed for phosphorus conversion. LB + LS treatment significantly increased total potassium and decreased available potassium, which demonstrated an enhancement of potassium utilization in rhizosphere soil. The generally increased availability of carbon and nutrients will also stimulate microbial activity, and this is corroborated by the increased levels of enzyme activity (Figure 5). Notably, the physicochemical properties of LB + HS rhizosphere soil did not differ significantly from the other treatments’ soil, except for HS, where both its sucrase (S-SC) and acid phosphatase (S-ACP) enzyme activities were significantly the highest and on par with those of HS. However, the urease (S-UE) enzyme tended to decline in activity after the addition of PGPM and sucrose. These results demonstrated that the co-effects of PGPM and sucrose in the LB + LS treatment did not lead to a significant net difference in the total contents of soil nutrients, but they significantly increased the contents of total potassium. This could have mediated the bioavailability of potassium and nutrients’ cycling in the soil.

### 2.4. The Effects of PGPM and Sucrose Applications on Rhizosphere Microbial Community Diversity and Richness

**Figure 6:**
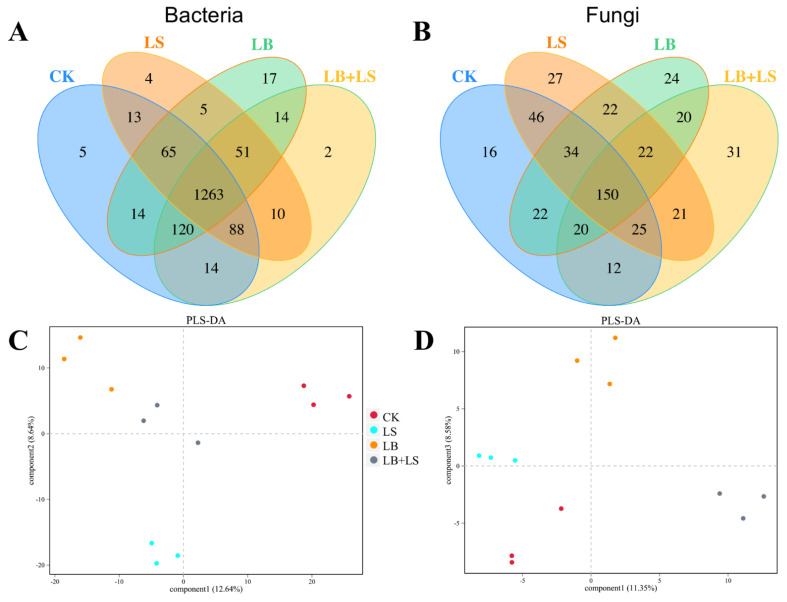
Diversity and richness of microbial communities in *R. palmatum* rhizosphere soil. Venn diagram depicting number and overlap OTUs of bacterial (**A**) and fungal (**B**) communities. Partial least squares discriminant analysis (PLS-DA) of bacterial (**C**) and fungal (**D**) communities. CK, water control; LS (low sucrose), 0.15 g/L of sucrose; LB (low *Bacillus*), 1.0 × 10^5^ CFU/mL of *Bacillus*; LB + LS, 1.0 × 10^5^ CFU/mL of *Bacillus* + 0.15 g/L of sucrose.

Profiles of 16S rRNA gene-based bacterial sequencing and ITS rRNA gene-based fungal sequencing were generated to better reveal the microbial community composition of rhubarb’s rhizosphere. This resulted in a total of 121,255 and 152,929 clean CCS reads across the 12 treatment samples in bacterial and fungal communities, respectively. The sequencing results are summarized in Supplementary Table S4, and they confirmed the reliability of the obtained sequence data volumes. Using a 97% nucleotide sequence identity, these sequences were grouped into 1685 bacterial OTUs and 492 fungal OTUs, with the CK rhizosphere having the greatest abundance of bacterial OTUs (1582), followed by LB + LS (1562), LB (1549), and LS (1499) treatments, whereas LS rhizosphere showed the highest fungal OTU numbers (347), followed by CK (325), LB (314), and LB + LS (301) treatments (Figure 6A,B). The Venn diagram depicts the unique and shared OTUs among treatments, which revealed that 5, 4, 17, and 2 OTUs were unique to the CK, LS, LB, and LB + LS treatments, respectively, with 1263 shared OTUs in bacterial communities. In fungal communities, 16, 27, 24, and 31 OTUs were unique to the CK, LS, LB, and LB + LS treatments, respectively, and 150 OTUs were shared (Figure 6A,B). Furthermore, the rarefaction curve of each sample approached a saturation plateau (Supplementary Figure S4), which also validated the good quality of our data.

The PLS-DA score plots showed that the samples from the four treatments (CK, LS, LB, and LB + LS) were well-separated in both the bacterial (12.64% and 8.64% of the total variables, respectively) and fungal communities (11.35% and 8.58% of the total variables, respectively) (Figure 6C,D), in that the positions (dots per group) were scattered far away from the other treatment groups. The alpha diversity indices of the soil microbial community are conveyed in Table 2. For bacteria, although the difference was not significant when compared to CK, both the richness indices (Ace and Chao1) and diversity indices (Simpson, Shannon, except PD whole tree) in the treatments were all reduced, especially in LS soil. However, there was a significant difference in the Simpson index between CK and LB + LS when analyzed by Student’s *t*-test (*p* = 0.0147, data not shown). For fungi, when compared to CK, the LS sustained higher richness (Ace and Chao1), while PGPM-containing treatments of LB + LS and LB had lower values for either index. Compared with CK, both Simpson and Shannon diversity indices were increased under all three treatments, whereas the PD whole tree index was significantly lower in LB + LS (Student’s *t*-test, *p* = 0.0808, data not shown). Therefore, we speculated that there were two consequences of our treatments: the sucrose-added LS reduced the richness and diversity of bacteria, and PGPM-included LB + LS and LB reduced that richness but induced a greater diversity of fungi.

### 2.5. The Effects of PGPM and Sucrose Applications on Rhizosphere Microbial Community Composition

**Figure 7:**
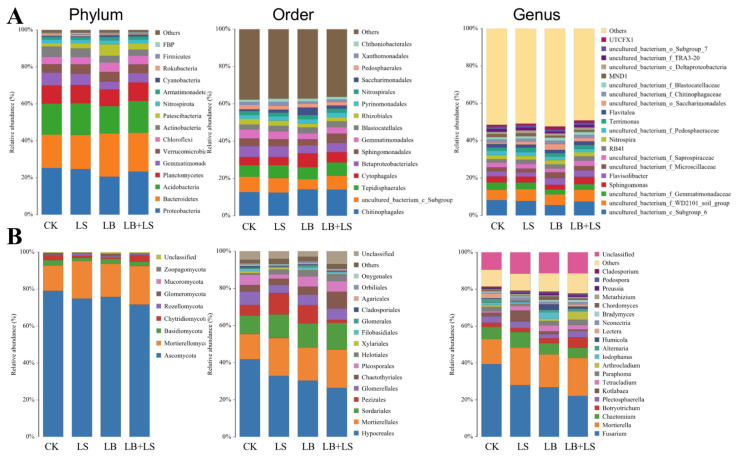
Relative abundances of bacterial (**A**) and fungal (**B**) communities in *R. palmatum*’ rhizosphere soil at the taxonomic level of phylum, order, and genus under the sucrose and PGPM treatments. CK, water control; LS (low sucrose), 0.15 g/L of sucrose; LB (low *Bacillus*), 1.0 × 10^5^ CFU/mL of *Bacillus*; LB + LS, 1.0 × 10^5^ CFU/mL of *Bacillus* + 0.15 g/L of sucrose.

Further analysis of microbial community structure indicated that the 16S rRNA gene sequences were affiliated with 31 phyla, 257 families, and 396 genera, while the ITS rRNA gene sequences were affiliated with 8 phyla, 100 families, and 170 genera. Figure 7 shows the 15 most abundant phyla and orders, and top 20 genera, found in the community composition. The *Proteobacteria*, *Bacteroidetes*, *Acidobacteria*, and *Planctomycetes* were the four most predominant bacterial phyla across all samples (Figure 7A), accounting for 67.8–71.68% of the relative abundances of all classified bacterial sequences (Supplementary Figure S5A). *Proteobacteria* was the dominant bacterial phylum in CK, LS, and LB +LS soils, while *Bacteroidetes* (23.4%) was the highest phylum in the LB-treated soil, followed by *Proteobacteria* (20.6%). This indicated that the bacterial community of soils after PGPM amendments was significantly different from that after sucrose treatment (LS) or in the CK. Moreover, the relative abundance of *Cyanobacteria* was increased in the LB- and LB + LS-treated samples, whereas *Rokubacteria* was increased in CK and LS samples. At the order level, *Chitinophagales*, followed by *uncultured_bacterium_c_Subgroup_6*, *Tepidisphaerales*, *Cytophagales*, *Betaproteobacteriales*, and *Sphingomonadales*, were the six most predominant bacteria present in soil across all samples. Among these six orders, the soil under the LB treatment sustained the highest level of *Cytophagales* and *Saccharimonadales* when compared to CK and LS. The soil of these two treatments not only harbored a similar bacterial composition at the order level but also at the genus level. In particular, relative abundance of the first dominant genus *uncultured_bacterium_c_Subgroup_6* was decreased while that of *uncultured_bacterium_o_ Saccharimonadales* was increased by the LB treatment with PGPM application to soil. In summary, our results indicated that sucrose addition had little impact on bacterial community structure in LS soil, yet it modulated the diversity induced by the PGPM addition in LB + LS soil.

Of the classified fungal community (Figure 7B), the *Ascomycota* and *Mortierellomycota* were the two most abundant phyla, together accounting for more than 92.5% of the relative abundance of the total fungal sequences (Supplementary Figure S5B). The most abundant phylum *Ascomycota* was enriched the most in CK (79.1%), and diminished the most in LB + LS (71.6%) treatment. In contrast, the generally second-ranked phylum *Mortierellomycota* was higher in abundance under the LB + LS treatment. Moreover, the third-most abundant phylum *Basidiomycota* seemed sensitive to sucrose addition. Interestingly, the relative abundance of *Chytridiomycota* was decreased in LS and LB treatments. At the order level, *Hypocreales*, followed by *Mortierellales*, *Sordariales*, *Pezizales*, *Glomerellales*, *Chaetothyriales*, and *Pleosporales*, were the seven most predominant fungi across all samples. Among them, *Mortierellales* and *Sordariales* reached higher levels, whereas *Hypocreales* decreased across all treatments in comparison to the CK. Notably, although *Pezizales* was increased in LS- and LB-treated soils, its abundance was unexpectedly the lowest under the LB + LS treatment, which surprisingly had the greatest abundance of *Chaetothyriales*. At the genus level, compared with CK, the first dominant genus *Fusarium* was decreased, whereas the second dominant genus *Mortierella* was increased in all treatments, with the highest increase recorded in the LB + LS treatment. Besides, each treatment evidently was able to enrich a specific genus; for instance, *Alternaria* and *Lectera* in CK, *Kotlabaea* in LS, *Iodophanus* and *Humicola* in LB, and *Botryotrichum* and *Arthrocladium* in LB + LS. In short, unlike the bacterial community, the fungal community structure changed distinctly between the CK vis-à-vis the three treatments, and even across the treatments.

### 2.6. The Effects of Soil Physicochemical Properties on the Microbial Abundances

**Figure 8:**
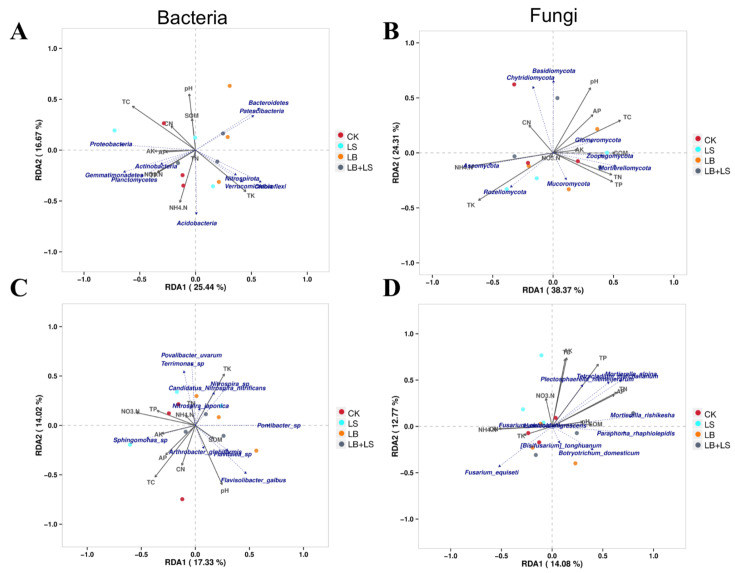
Redundancy analysis (RDA) plots of microbial communities at the taxonomic level of phyla (**A**,**B**) and species (**C**,**D**), and selected soil physiochemical properties under sucrose and PGPM treatments. SOM, soil organic matter; TC, total carbon; TN, total nitrogen; C/N, carbon to nitrogen ratio; NH_4_-N, ammonium nitrogen; NO_3_-N, nitrate nitrogen; AP, available phosphorus; AK, available potassium; TP, total phosphorus; TK, total potassium. CK, water control; LS (low sucrose), 0.15 g/L of sucrose; LB (low *Bacillus*), 1.0 × 10^5^ CFU/mL of *Bacillus*; LB + LS, 1.0 × 10^5^ CFU/mL of *Bacillus* + 0.15 g/L of sucrose.

We also performed redundancy analysis (RDA) based on the selected soil physicochemical properties and top 10 microbial abundances at the phyla and species levels (Figure 8). The results showed that the first and second RDA components could explain 42.11% (phyla) and 31.85% (species) of the total variation in bacteria, and likewise 62.68% (phyla) and 26.85% (species) of the total variation in fungi. Examined at the phylum level, the structure of the total bacterial communities was strongly correlated with certain soil physicochemical properties: TC, TK, PH, and NH_4_-N (Figure 8A). The LS soil was dominated by the *Proteobacteria*, *Gemmatimonadetes*, and *Planctomycetes,* which were most associated with TC and NH_4_-N, the LB-treated soil was dominated by the *Bacteroidetes*, *Patescibacteri*, and *Verrucomicrobia*, and these were most associated with TK and pH, while the LB + LS soil was dominated by the *Verrucomicrobia*, *Chloroflexi*, and *Acidobacteria*, that were all associated with soil TK and NH_4_-N. Accordingly, the relationships between soil physicochemical properties and relative abundances of the dominant bacterial species revealed pH, TC, NO_3_-N, and TK as the influential environmental factors (Figure 8C). The RDA results also suggested that *Nitrospira* and *Pontibacter* had relative abundances that were associated with TK content, but inversely related to TC in the LB-treated soil. Relative abundances of *Flavisolibacter* and *Pontibacter* were both associated with pH in the LB + LS soil, whereas *Sphingomonas* had relative abundances associated with AK and TC.

For the fungi at the phylum level, total community composition was strongly correlated with the following soil physicochemical properties: TK, NH_4_-N, TC, pH, and TP (Figure 8B). The LS soil was dominated by the *Rozellomycota* and *Mortierellomycota*, whose members were associated most with TK and TP, the LB-treated soil was dominated by the *Mortierellomycota*, *Zoopagomycota*, and *Mucoromycota*, that were associated most with TP, TN, and SOM, and the LB + LS soil was dominated by both *Basidiomycota* and *Chytridiomycota*, and these were associated with soil pH and C/N. Accordingly, the relationships between soil physicochemical properties and relative abundances of dominant fungi at the species level revealed that the main environmental factors influencing the community were TP, TN, AP, AK, TC, and NO_3_-N (Figure 8D). The RDA results also suggested that *Fusarium_equiseti* abundance was associated with TK and the NO_3_-N content in both CK and the LB-treated soil, *Fusarium_domesticum* and *Humicola_nigrescens* had relative abundances that were associated with the NO_3_-N content under the LS treatment, and for *Paraphoma_rhaphiolepidis*, *Botryotrichum_domesticum*, and *Mortierella_rishikesha*, their relative abundances were associated with SOM and pH in the LB + LS soil.

### 2.7. Network Analysis and Effects of PGPM and Sucrose Applications on Rhizosphere Microbial Community Function

**Figure 9:**
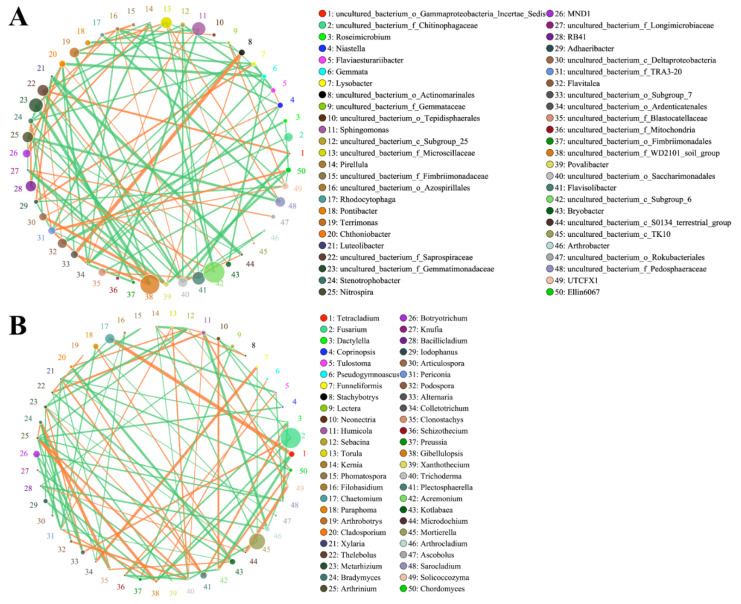
Spearman’s correlation-based network analysis of bacterial (**A**) and fungal (**B**) communities for interactions among genera. Nodes (colored dots) are genera, whose size is proportional to their relative abundance. A line between two nodes indicates a significant positive (orange, Spearman’s correlation, r_s_ > 0.1 and *p* < 0.05) or negative (green, Spearman’s correlation, r_s_ > −0.1 and *p* < 0.05) correlation, with its thickness reflecting the relative strength.

To further elucidate the possible ‘collaborative’ or ‘competing’ relationships among different microbial communities in the rhizosphere, Spearman’s rank correlation coefficients between the most abundant genera were derived (Figure 9). The network exhibited correlations among the 50 dominant bacterial and fungal genera. For bacteria, *uncultured_bacterium_o_Actinomarinales* and *uncultured_bacterium_o_Subgroup_7* were the most positively correlated genera (r_s_ = 0.42), while in the fungal community network, the most positively correlated genera were *Chaetomium* and *Tetracladium* (r_s_ = 0.42).

The Clusters of Orthologous Groups of proteins (COG) functional classification was used to predict both functional distribution and abundance based on the 16S rRNA gene soil bacterial composition data (Supplementary Figure S6). When compared with CK soil, we found 18, 23, and 17 pathways, respectively, enriched by the LS, LB, and LB + LS treatments. Among these comparisons, amino acid transport and metabolism, translation, ribosomal structure and biogenesis, cell wall/membrane/envelope biogenesis, and inorganic ion transport and metabolism were significantly enriched in the LS soil samples. Under the LB treatment, coenzyme transport and metabolism, signal transduction mechanisms, and nucleotide transport and metabolism were significantly enriched. Finally, carbohydrate transport and metabolism, replication, recombination, inorganic ion transport and metabolism, post-translational modification, protein turnover, and chaperones were all significantly enriched in the LB + LS samples.

## 3. Discussion

Relying on PGPM inoculants to enhance plant fitness and soil health is quickly establishing itself as a viable strategy for sustainable agriculture. A case in point is *R. palmatum*, a high-altitude endangered medicinal plant for which PGPM inoculants could be an ideal tool, especially given its economic benefits for herb farmers. In this study, the *Bacillus* strain EZ99 was tested for its potential to promote plant growth of *R. palmatum*, added with different concentrations of sucrose. The experimental results revealed differential impacts on key growth and development parameters: plant height, crown width, leaf length and width, root weight and length, taproot diameter, and lateral root numbers. In particular, the treatments LB + LS and LB + MS significantly increased most of these growth and yield parameters (Figure 1 and Figure 2). A single application of PGPM, such as *B. amyloliquefaciens* FZB42, at a concentration above 1.0 × 10^5^ CFU/mL, has been shown capable of substantially promoting the growth of diverse plants, such as *Arabidopsis thaliana*, tomato, maize, and wheat, via enhanced photosynthesis [49,50,51,52,53,54]. Sucrose is a common photosynthate that plants produce for their own metabolism or which is secreted as root exudates for host colonization by microbes. The addition of a high level of sucrose (15 g/L) to *A. thaliana* [55] and potato [56] generated a plant phenotype distinguished by later onset of flowering and more leaves, which was due to the regulation of sets of sucrose-metabolizing enzymes such as invertase (INV) [57], sucrose synthase (Sus) [58], and the sucrose non-fermenting-regulating-related kinase (SnRK1) [59]. Furthermore, sucrose can also function as an osmotic signal to activate transcription factors, such as MYB75, phytochrome-interacting factor (PIF), and basic helix-loop-helix (bHLH), in plant cells, thereby affecting the growth, flowering [60], and productivity of plants and their circadian clock [61]. In our case, compared to the sole use of sucrose, administering PGPM coupled with a high sucrose concentration (LB + HS, MB + HS, and HB + HS) slowed both the aboveground and belowground growth of *R. palmatum* throughout the observation period of the experiment. This unexpected result could be explained by the countervailing effects of sucrose and PGPM, whereby sucrose served as a universal and indispensable carbon resource to all microbes in the rhizosphere, not only for *Bacillus*, although high sucrose concentrations (HS and LB + HS) significantly increased the soil S-SC activity and Microbial biomass carbon (MBC) (Figure 5).

The results from our widely targeted metabolomics approach provide strong support for a likely metabolic redirection ensuing in *R. palmatum* after its PGPM inoculant amendment with sucrose (Figure 4). Although various reports have characterized the mass spectra of diverse compounds harbored by rhubarb plants, few of them have comprehensively identified and differentiated these compounds. Many secondary metabolites derive from multiple branches of the phenylpropanoid pathway, including flavonoid, phenylpropanoids, phenolic compounds, and other anthocyanin compounds, and they are considered as crucial components of developmental and defense process [62]. Relative to CK, the LB + LS-treated rhubarb had the greatest number of DEMs. Anthraquinones, the major bioactive constituents of rhubarb, were enriched across the three selected treatments (LS, LB, and LB + LS) when compared to the control (CK), with only the enrichment of particular anthraquinones, which means that the treatments applied somehow reprogrammed the metabolism of anthraquinones. A similar pattern also appeared for flavonoids, in that they were downregulated, whereas phenolic compounds were upregulated in rhubarbs under LB + LS, in contrast to results reported for cases where sucrose and bacteria were applied separately to plants [63,64]. This suggests that the synthesis of flavonoids triggered by sucrose and bacteria applied separately can be redirected to phenolic compounds by their joint application [65]. Moreover, for carbohydrates, our findings uncovered a global accumulation of saccharides in the LS and LB treatment samples, but a downregulation of organic acids occurred in the LB + LS treatment sample. To fully discern the differing patterns of metabolite accumulation between combined (LB + LS) and separated (LS and LB) applications of sucrose and bacteria inoculants, supplementary analytical tools (such as transcriptome profiling in situ) are needed to further explore and elucidate the involved mechanisms.

Many studies have investigated how soil physicochemical properties and microbial community composition influence extracellular enzyme activities with respect to the regulation of extracellular enzyme levels [66,67,68]. Our study also investigated the impact of external applications of sucrose, *Bacillus* inoculant, and their mixed form upon rhizosphere soil and bulk soil, to assess how the physicochemical composition of the two soils responded to the treatments (Table 1). Although the changes among treatments were negligible for most variables, we did find that the AP and TP were significantly increased in the HS rhizosphere soil. These results suggest that the carbon supplied by the addition of a high sucrose concentration (15 g/L) contributed to the conversion of phosphorus and nutrients, with the PGPM addition accelerating phosphorus utilization, thereby stimulating S-SC, S-ACP, and S-ALP enzyme activities, except that of S-UE (Figure 5). Soil enzymes dominated the soil biochemical processes; hence, increased enzyme activities imply strengthened microbial metabolic activity and soil carbon and phosphorus use [69,70]. Besides, *Bacillus* has been proven to possess the ability to dissolve K from K-minerals and enhance plant growth and yield [71]. Although the content of AK and TK remained stable under separately applied sucrose and bacteria, LB + LS treatment significantly increased TK, whereas it decreased AK in rhizosphere soil, implying the co-effect of sucrose and bacteria in efficient potassium uptake. Another important component of soil quality is nitrogen, for which the nitrate (NO_3_^−^) form of bulk soil is greatly increased by lower soil pH [72]. In most cases, changes in soil physicochemical properties can also directly or indirectly influence the growth and development of plants and overall crop production.

Microbial communities and their composition can vary structurally in response to microbial inoculant applications [73]. Indeed, we found that the diversity and abundance of both bacteria and fungi in *Bacillus*-containing treatments differed the most from the control group (Figure 7 and Table 2). There is evidence that the introduction of biocontrol agents can drive shifts in rhizosphere microbial community structure [74,75,76]. Here, in the LB-treated sampled soil, *Bacteroidetes* was the dominant phylum, whereas *Proteobacteria* dominated the CK, LS, and LB + LS soils, which supports the hypothesis that sucrose present at a low concentration acts as a signal, with sucrose frequently being a chemotactic substance for rhizobacteria colonization, thus driving plant–soil feedbacks by shaping the rhizosphere microbiota. Recently, Mhlongo et al. [77] reported that the recruitment activity of root exudates for certain plant growth-promoting microbes diminished soil microbial diversity in the rhizosphere. However, in line with Mhlongo et al. [78], we found that the relative abundance of the genus *Bacillus* itself was comparatively lower, leaving it undetectable as a representative genus despite its external addition, probably because of niche competition with other rhizosphere bacteria fostering a boost in the abundance of *Bacteroidetes* bacteria [79]. Concerning the soil fungal community, a pronounced reduction in the *Fusarium* genus that contains many agronomically important pathogens [80] was observed among the three treatments, but especially in the LB + LS group, which illustrates the joint effect of sucrose and the inoculant. It is unsurprising that the combined application of PGPM with biochar has been proven more efficient than a single treatment alone in suppressing tomato early blight disease [81]. Besides, the added sucrose attracted the sugar-loving fungus *Mortierella*, an oleaginous fungus that can produce lipids to promote growth [65,82]. Meanwhile, in our study, each treatment enriched its own specific fungal genus, such as *Alternaria* [83], *Kotlabaea* [84], and *Botryotrichum* [85], for which some isolates are pathogenic, while these genera were diminished in other groups. This implies the inhibition of specific fungi by different treatments of sucrose, the inoculant, or their combination.

Finally, the RDA and Spearman’s rank correlation coefficients revealed a positive relationship between *Sphingomonas* and AK in the LB + LS treatment soil (Figure 8 and Figure 9). Considering the above results, we suggest that the co-occurring effects of low sucrose and *Bacillus* inoculant increased *R. palmatum’s* biomass and its medicinal ingredients, and improved its nutrient uptake from soil, which might in turn generate a signature microbial composition. In our study, the application of bacterial inoculant combined with sucrose promoted a significant increase in plant biomass and the reprogramming of secondary metabolites through a direct mechanism, and promoted soil nutrient uptake and biocontrol for causative pathogens via an indirect mechanism.

## 4. Materials and Methods

### 4.1. Plants, Bacteria, and Experiment Design

*R. palmatum* seedlings were purchased from a local seed distributor (Caoping town, Li County, Longnan city, Gansu province, China (33.40° N, 104.48° E; elevation: 2000 m)). They were used for the field trial experiment carried out at Gansu Gaolan Field Scientific Observation and Research Station for Agricultural Ecosystem (Zhonghe town, Gaolan county, Lanzhou city, Gansu province, China (38.22° N, 103.80° E; elevation: 1800 m)) on 28 April 2020. A 2-year vacant field previously cultivated with potato and without *R. palmatum* was selected for a ‘clear’ soil background. Seedlings were selected free of wounds and rot, and as uniform as possible in size (ca. 20 cm in length).

As the microbial inoculant, we used *B. amyloliquefaciens strain* EZ99 (GenBank accession number NZ_LPUP00000000), a Gram-positive bacterium originally isolated from bottom silt of the Baohai Sea, China. Briefly, EZ99 was grown in Luria–Bertani broth [86] at 37 °C, with shaking at 200 rpm in an orbital shaker for 16 h. Then, its cells were collected by centrifugation at 5000× *g* for 10 min, and washed with sterile water, to yield a final concentration of colony-forming units (CFU) ≥ 1.0 ×10^7^/mL as the inoculant.

A total of 16 treatments were applied. Three concentrations of *B. amyloliquefaciens* EZ99 inoculant (1.0 × 10^5^, 1.0 × 10^6^, and 1.0 × 10^7^ CFU/mL, denoted here by LB, MB, and HB, respectively) and sucrose (0.15, 1.5, and 15 g/L, denoted as LS, MS, and HS, respectively) were combined as follows: water control (CK), bacterial inoculant only (LB, MB, SB), sucrose only (LS, MS, HS), and bacterial inoculant + sucrose combinations (LB + LS, LB + MS, LB + HS, MB + LS, MB + MS, MB + HS, HB + LS, HB + MS, and HB + HS). The field experiment was set up as a randomized complete block (RCB) design with three plot replicates per treatment. Prior to the transplantation, the field was loosened using a rotary tiller. A row maker was then used to dig out narrow rows (20 cm width, 30 cm depth), with row spacing of 70 cm in the soil bed. Each plot was 10.5 m^2^ (3.5 × 3 m) with 50 (5 × 10) seedlings transplanted manually into the rows with plant spacing of 50 cm. About 1 month after transplantation, seedlings (*n* = 2400) were root-inoculated with the 1 L treatment as soil drench per plant, and applied again after 2 weeks. Throughout the growth stage of the experiment, management practices were uniformly implemented by following those recommended for this semi-arid region, which included weeding and irrigation as required.

### 4.2. Growth Parameters and Yield Analysis

Empirical measurements began 3 weeks after treatments were applied, and were performed monthly to quantify changes in plant height (cm), crown width (cm), leaf length (cm), and leaf width (cm). For these response variables, in each of the three replicate plots per treatment, at least 30 plants were randomly selected. After the harvest in November 2020, the yield parameters of roots—the taproot length (cm), total root length (cm), taproot diameter (cm), the number of lateral roots, fresh root weight (g), and dry root weight (g)—were also measured. In addition, the harvested roots and soils were collected, immediately frozen in liquid nitrogen, and transported to the laboratory. Then, the root and soil samples were each divided into two parts. One part of the root and soil samples was stored in dry ice and immediately sent to Biomarker Technologies Corporation (Beijing, China) for metabolome and microbiome analyses, respectively. The other part of root samples was stored at −80 °C (for metabolome). The remaining part of soil samples (rhizosphere soil and bulk soil) was again divided into two parts. One part was stored at –20 °C (for microbiome), and the other part was used for determining the physicochemical properties, enzyme activities, and microbial biomass of soil (stored at 4 °C). Three replicates were used for each analysis, except six replicates for microbial biomass.

### 4.3. Determination of Soil Physicochemical Properties, Enzyme Activities, and Microbial Biomass

Soil samples were air-dried and sifted through a 1 mm sieve, then thoroughly homogenized. The pH level was measured with a DELTA320 pH meter (Mettler Toledo, Greifensee, Switzerland) using a 1:1 air-dried soil to distilled water ratio. Soil water content (SWC) was measured by the gravimetric method. Soil organic matter (SOM) was quantified using the K_2_Cr_2_O_7_-H_2_SO_4_ oxidation method and a digital burette (Brand Titrette, Wertheim, Germany) [87]. Total carbon (TC) and total nitrogen (TN) were each determined by a TOC-V CPH total organic carbon analyzer (Shimadzu, Tokyo, Japan), from which the carbon to nitrogen ratio (C/N) of soil was then calculated. Both soil ammonium nitrogen (NH_4_-N, AN) and nitrate nitrogen (NO_3_-N, NN) contents were extracted with 1 M KCl and detected by a flow injection analyzer (AutoAnalyzer AA3, Seal Systems, Norderstedt, Germany). Total phosphorus (TP) was determined by the sodium hydroxide melting-molybdenum antimony colorimetric method, and available phosphorus (AP) was determined by the 0.5 M sodium bicarbonate extraction-molybdenum antimony colorimetric method, with a UV-2450 spectrophotometer (Shimadzu, Tokyo, Japan) [88]. Total potassium (TK) was determined by sodium hydroxide melting-flame spectrophotometry, and available potassium (AK) was determined by 1 M ammonium acetate extraction-flame spectrophotometry, using a FP6450 flame photometer (Inesa, Shanghai, China) [89].

Fresh rhizosphere soil was used to study soil enzyme activities. Specifically, the soil sucrase (S-SC, EC3.2.1.26), urease (S-UE, EC3.5.1.5), acid phosphatase (S-ACP, EC 3.1.3.2), and alkaline phosphatase (S-AKP, EC3.1.3.1), which are involved in the degradation of carbon, nitrogen, and phosphorus, were selected. Commercial testing kits (Comin Biotechnology, Suzhou, China) and spectrophotometry were used accordingly to determine the activity levels of the four enzymes in soil [90].

Microbial biomass carbon (MBC) was used here as a proxy for soil microbial biomass (SMB). MBC was determined by the chloroform fumigation-extraction method [91]. Briefly, fresh soil samples equivalent to 20 g of air-dried soil were fumigated with alcohol-free CHCl_3_ at 25 °C, for 24 h, then C was extracted from the fumigated and non-fumigated samples with 0.5 mol L^−1^ K_2_SO_4_ (soil:water = 1:4 (*w*/*v*)) for 1 h. These filtered extracts were analyzed using a TOC-V CPH total organic carbon analyzer (Shimadzu, Tokyo, Japan). The conversion factor, k_EC_, between fumigation and un-fumigation was fixed at 0.45 [92,93].

### 4.4. Metabolomic Profiling

The metabolites’ extraction and widely targeted metabolomics were both performed by the Biomarker Technologies Corporation (Beijing, China). In brief, rhubarb root tissue samples were ground in liquid nitrogen and freeze-dried, then crushed with a zirconia bead at 30 Hz for 1.5 min in a MM 400 mixer mill (Retsch, Haan, Germany). From each sample, 100 mg of powder was extracted with 1.0 mL of 70% aqueous methanol—containing 0.1 mg/L lidocaine as an internal control and kept overnight at 4 °C. After centrifugation at 10,000× *g* for 10 min, the sample extracts were absorbed using CNWBOND Carbon-GCB SPE Cartridge (250 mg/3 mL; Anpel, Shanghai, China) and filtrated using a SCAA-104 syringe filter (0.22 μm pore size; Anpel, Shanghai, China), and all of them were stored individually in glass vials for the analyses below.

The extracts were analyzed by a liquid chromatography-electrospray ionization-tandem mass spectrometry (LC-ESI-MS/MS) system (high-performance liquid chromatography (HPLC): Shim-pack UFLC Shimadzu CBM30A system; MS, Applied Biosystems 6500 Q TRAP). The analytical conditions were as follows: HPLC column, Waters ACQUITY UPLC HSS T3 C18 (1.8 µm, 2.1 × 100 mm); solvent system, water (0.04% acetic acid):acetonitrile (0.04% acetic acid); gradient program, 95:5 *v*/*v* at 0 min, 5:95 *v*/*v* at 11 min, 5:95 *v*/*v* at 12 min, 95:5 *v*/*v* at 12.1 min, and 95:5 *v*/*v* at 15 min; temperature, 40 °C; flow rate, 0.40 mL/min; injection volume, 2 μL. The effluent was connected to an ESI-triple-quadrupole-linear ion trap (QTRAP)-MS.

Triple-quadrupole (QQQ) scans and linear ion trap (LIT) were acquired on a Q- TRAP mass spectrometer (API 6500 Q TRAP LC/MS/MS System), equipped with an ESI Turbo Ion-Spray interface, operating in a positive ion mode and controlled by SCIEX Analyst software. The ESI source operation parameters were set as follows: ion source, turbo spray; ion spray voltage, 5500 V; source temperature, 500 °C; ion source gas I, gas II, and curtain gas of 55, 60, and 25.0 psi, respectively; the collision activated dissociation was set at “high”. Instrument tuning and mass calibration were performed with 10 and 100 μmol/L of polypropylene glycol solutions in the QQQ and LIT modes, respectively. Metabolites’ identification was based on the self-built database (http://www.biomarker.com.cn/) (accessed on 20 January 2022) and several publicly available metabolite databases, namely HMDB (http://www.hmdb.ca/), MassBank (http://www.massbank.jp/) (accessed on 20 January 2022), MoToDB (http://www.ab.wur.nl/moto/) (accessed on 5 January 2021), and METLIN (http://metlin.scripps.edu/index.php) (accessed on 20 January 2022). The QQQ scans were acquired as multiple reaction monitoring (MRM) experiments, with nitrogen as the collision gas set to 5 psi [94]. Collision energy (CE) and de-clustering potential (DP) for individual MRM transitions were carried out by the optimization of CE and DP. A specific set of MRM transitions was monitored for each period according to the metabolites eluted within the period.

Partial least squares discriminant analysis (PLS–DA) and orthogonal projections to latent structures discriminant analysis (OPLS-DA) were used to determine differentially expressed metabolites (DEMs). The relative importance of each metabolite to the OPLS-DA model was checked using the variable importance in projection (VIP) metric. Metabolites that met the threshold criteria of VIP ≥ 1, fold change ≥ 1.5, and *p*-value ≤ 0.05 were designated as differentially expressed metabolites (DEMs). These annotated metabolites were mapped to the Kyoto Encyclopedia of Genes and Genomes (KEGG) for pathway enrichment analysis, using the R package v3.3.2 ‘clusterProfiler’ [95].

### 4.5. Bacterial and Fungal PacBio Sequencing

To characterize the composition of potentially microbial communities in the rhizosphere samples of the treatments, bacterial and fungal profiling was carried out by the Biomarker Technologies Corporation (Beijing, China) (http://www.biomarker.com.cn/) (accessed on 20 January 2022). Microbial genomic DNA (from active or dormant cells) was extracted using the PowerSoil DNA Isolation Kit (Mo Bio, Solana Beach, CA, USA). This was subjected to high-throughput sequencing targeting the V4 regions of the 16S rRNA gene of bacteria, using the universal primer pair 27F (5′-AGRGTTYGATYMTGGCTCAG-3′) and 1492R (5′-RGYTACCTTGTTACGACTT-3′), while the fungal internal transcribed spacer (ITS) region ITS1 was targeted with the universal primer pair ITS1F (5′-CTTGGTCATTTAGAGGAAGTAA-3′) and ITS1R (5′-TCCTCCGCTTATTGATATGC-3′), with specific barcodes included, via PCR. The quality and concentration of the ensuing PCR products were assessed using a NanoDrop spectrophotometer (Thermo Scientific, Waltham, MA, USA). Then, the extracted DNA was used to build a genomic library and sequenced on the single-molecule real-time (SMRT) sequencing platform by the PacBio RS II system (Pacific Bioscience, Menlo Park, CA, USA). The total circular consensus sequences (CCS) were generated and used for further downstream analyses, which entailed filtering by lima v1.7.0 and cutadapt v1.9.1 tools [96], and checking for any chimeras with UCHIME v4.2 [97]. The remaining sequences were classified by USEARCH into taxa by BLAST, each against the Ribosomal Database Project (RDP) database at a 97% similarity threshold [98]. Clustering of operational taxonomic units (OTUs) was performed and retained for further analysis [99]. The levels of alpha diversity (Ace, Chao1, Simpson, Shannon, and PD whole tree) and beta diversity (PLS-DA) were assessed by the QIIME pipeline [100]. The functional genes’ prediction was performed using PICRUSt and referencing the COG database [101]. RDA analysis was carried out using RDA/CCA tools on the platform BMKCloud (www.biocloud.net) (accessed on 20 January 2022). A network layout of microbial communities showing the interactions among genera based on their Spearman correlation matrix was implemented in Python [102]. All the raw data have been deposited in the NCBI Sequence Read Archive (SRA) database under accession number PRJNA755832.

### 4.6. Statistical Analysis

Each experiment was performed independently three times. The data were analyzed with SAS v9.4 software (SAS, Cary, NC, USA) or GraphPad Prism 9. All the values are expressed as the mean ± standard error (SE). Student’s *t*-test was conducted to determine the statistical differences between two parameters of mean values. One-way analysis of variance (ANOVA) was followed by Tukey’s HSD (honestly significant difference) test to determine the statistical differences between more than two parameters via multiple pairwise comparisons of their mean values.

## 5. Conclusions

This study demonstrated that the novel approach of co-applying sucrose and a bacterial inoculant is capable of significantly promoting the quantity and quality of *R. palmatum* and stimulating rhizosphere priming. We anticipated its promising application in plant microecology, which contributed to the sustainability of agricultural systems at lower production costs. The underlying mechanism should now be further sought and elucidated in a model plant, in a project that we have already begun. Another important knowledge gap to fill is to understand the potential of diverse bacterial consortia.

## Figures and Tables

**Table 1:** Soil physicochemical properties following the application of sucrose and the PGPM inoculant.

Treatment	pH	SWC	SOM	TC	TN	C/N	NO_3_-N	NH_4_-N	AP	AK	TP	TK
		(%)	(g/kg)	(g/kg)	(g/kg)	(mg/kg)	(mg/kg)	(mg/kg)	(mg/kg)	(mg/kg)	(g/kg)	(g/kg)
CK	8.5	13.55 ± 2.59	16.77 ± 0.33 ab	24.64 ± 0.43	1.05 ± 0.05	23.66 ± 1.41	14.65 ± 1.03 b	10.29 ± 1.17	30.25 ± 2.73 bc	321.67 ± 58.54 a	1.04 ± 0.03 ab	19.01 ± 0.08 b
LS	8.6	12.34 ± 1.31	16.87 ± 0.38 ab	25.14 ± 1.17	1.10 ± 0.01	22.84 ± 1.30	13.70 ± 1.08 b	9.55 ± 1.15	26.25 ± 2.19 cd	173.33 ± 17.53 b	1.07 ± 0.02 ab	19.17 ± 0.14 ab
HS	8.6	14.51 ± 1.58	18.04 ± 0.90 a	25.37 ± 0.37	1.17 ± 0.10	22.00 ± 1.91	12.12 ± 1.18 b	7.70 ± 0.42	39.85 ± 3.20 a	232.00 ± 15.95 b	1.15 ± 0.05 a	19.38 ± 0.11 a
LB	8.6	13.90 ± 2.01	16.96 ± 1.82 ab	24.24 ± 0.63	0.98 ± 0.06	24.93 ± 1.59	10.26 ± 0.40 b	8.61 ± 0.63	20.08 ± 2.14 b	226.33 ± 15.21 b	0.97 ± 0.02 b	19.12 ± 0.11 ab
LB + LS	8.6	14.88 ± 0.27	16.14 ± 1.13 ab	24.38 ± 0.53	1.09 ± 0.09	22.80 ± 1.65	11.65 ± 0.27 b	8.82 ± 0.50	34.45 ± 3.34 b	228.67 ± 0.33 b	1.04 ± 0.05 ab	19.10 ± 0.02 a
LB + HS	8.7	14.28 ± 1.57	14.81 ± 0.92 b	24.99 ± 0.91	1.06 ± 0.12	23.91 ± 1.88	10.05 ± 0.94 b	7.78 ± 0.13	23.22 ± 1.77 b	321.67 ± 58.54 a	1.00 ± 0.04 b	19.09 ± 0.09 ab
Bulk soil	8.5	13.82 ± 1.59	14.95 ± 0.08 b	24.86 ± 0.04	0.99 ± 0.03	25.35 ± 0.85	33.19 ± 2.98 a	6.43 ± 1.37	23.38 ± 1.08 b	173.33 ± 17.53 b	1.00 ± 0.02 b	19.13 ± 0.08 ab

Note: SWC, soil water content; SOM, soil organic matter; TC, total carbon; TN, total nitrogen; C/N, carbon to nitrogen ratio; NH_4_-N, ammonium nitrogen; NO_3_-N, nitrate nitrogen; AP, available phosphorus; AK, available potassium; TP, total phosphorus; TK, total potassium. CK, water control; LS (low sucrose), 0.15 g/L of sucrose; HS (high sucrose), 1.5 g/L of sucrose; LB (low *Bacillus*), 1.0 × 10^5^ CFU/mL of *Bacillus*; LB + LS, 1.0 × 10^5^ CFU/mL of *Bacillus* + 0.15 g/L of sucrose; LB + HS, 1.0 × 10^5^ CFU/mL of *Bacillus* + 1.5 g/L of sucrose. Values are the mean ± SE (*n* = 3). Different lowercase letters indicate a significant difference at *p* ≤ 0.05. Absence of lowercase letters indicates that differences were non-significant.

**Table 2:** Diversity of the microbial communities in *R. palmatum* rhizosphere soil.

Treatment	16S rRNA Gene-Based Bacteria					ITS rRNA Gene-Based Fungi				
	ACE	Chao1	Simpson	Shannon	PD Whole Tree	ACE	Chao1	Simpson	Shannon	PD Whole Tree
CK	1482.56 ± 23.88	1459.98 ± 23.60	0.9966 ± 0.0001	9.11 ± 0.04	71.93 ± 2.28	211.57 ± 12.60	209.90 ± 10.78	0.8651 ± 0.05	4.46 ± 0.50	36.50 ± 0.44
LS	1384.83 ± 60.32	1341.31 ± 67.73	0.9958 ± 0.0003	8.87 ± 0.12	63.74 ± 6.85	213.19 ± 10.85	213.63 ± 10.31	0.8900 ± 0.03	4.68 ± 0.25	36.60 ± 1.36
LB	1464.27 ± 40.35	1410.03 ± 30.48	0.9960 ± 0.0003	8.97 ± 0.06	72.17 ± 2.77	183.84 ± 19.97	182.62 ± 19.96	0.9201 ± 0.03	5.00 ± 0.33	33.84 ± 2.52
LB + LS	1463.71 ± 23.12	1409.60 ± 17.86	0.9962 ± 0.0001	9.02 ± 0.01	70.53 ± 1.78	171.36 ± 11.86	171.17 ± 12.00	0.9437 ± 0.02	5.32 ± 0.22	32.08 ± 1.47

Note: CK, water control; LS (low sucrose), 0.15 g/L of sucrose; LB (low *Bacillus*), 1.0 × 10^5^ CFU/mL of *Bacillus*; LB + LS, 1.0 × 10^5^ CFU/mL of *Bacillus* + 0.15 g/L of sucrose. Values are the mean ± SE (*n* = 3). Absence of lowercase letters indicates that differences were non-significant at *p* ≤ 0.05.

## Data Availability

The raw bacterial 16S and fungal ITS sequence data have been deposited at the NCBI Sequence Read Archive (SRA) database under accession number PRJNA755832.

## Supplementary Materials

The following are available online at https://www.mdpi.com/article/10.3390/ijms23031694/s1.
