# Supplementary material for: Bacterial Inoculant and Sucrose Amendments Improve the Growth of Rheum palmatum L. by Reprograming Its Metabolite Composition and Altering Its Soil Microbial Community
Source: Int J Mol Sci. 2022 Feb 1;23(3):1694. doi: 10.3390/ijms23031694 (PMC8835959; doi:10.3390/ijms23031694)
Supplement: Supplementary file 1 [file ijms-23-01694-s001.zip › Supplementary Materials/Supplementary Material-IJMS-TY.pdf]

Supplementary Material

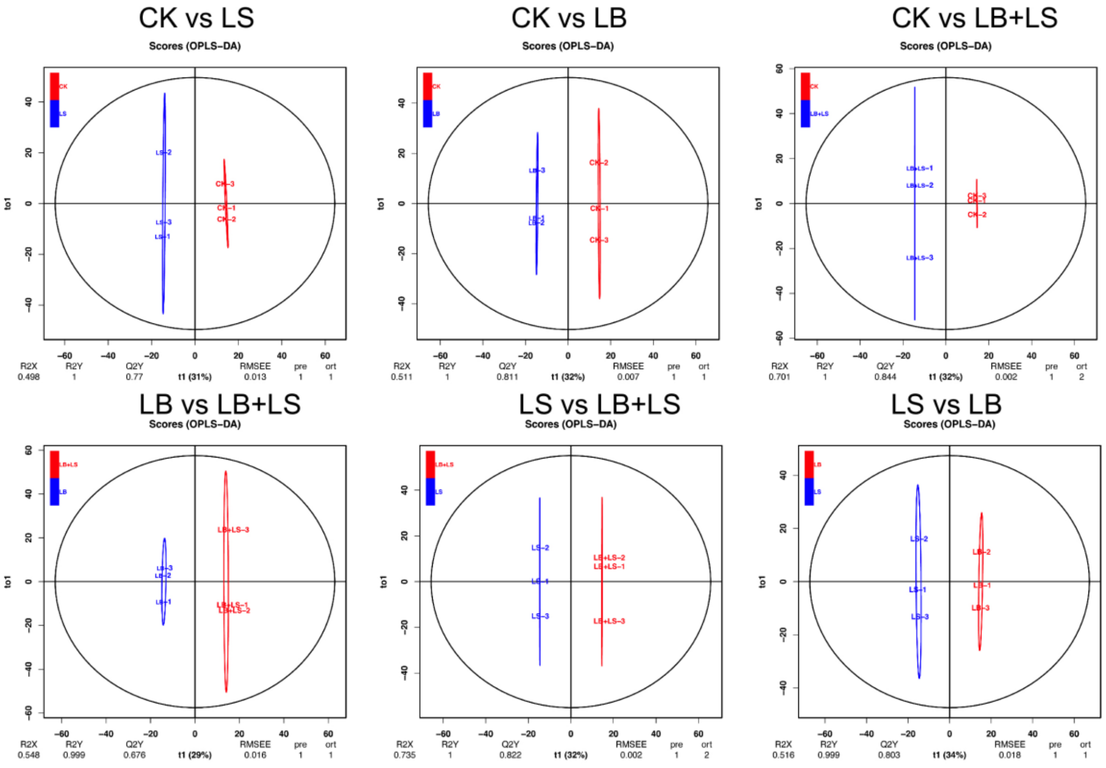

Figure S1. Orthogonal partial least squares-discriminant analysis (OPLS-DA) of metabolic profiles in each comparison.

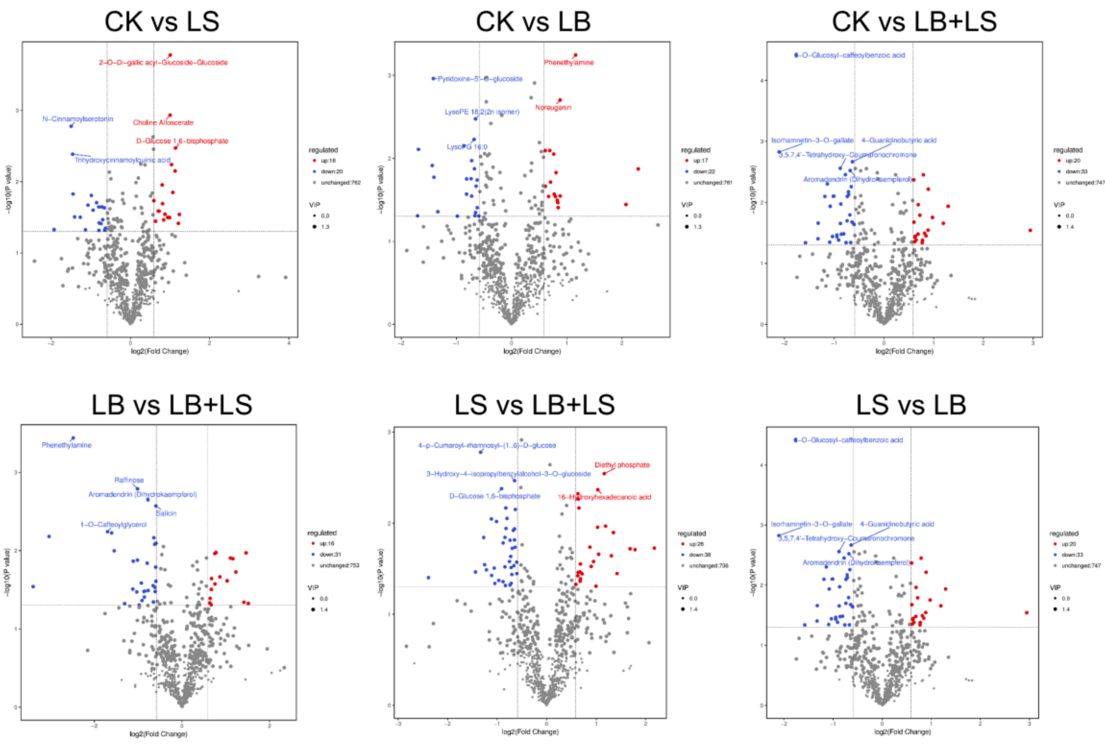

Figure S2. Volcano plots of differentially expressed metabolites (DEMs) identified in each comparison.

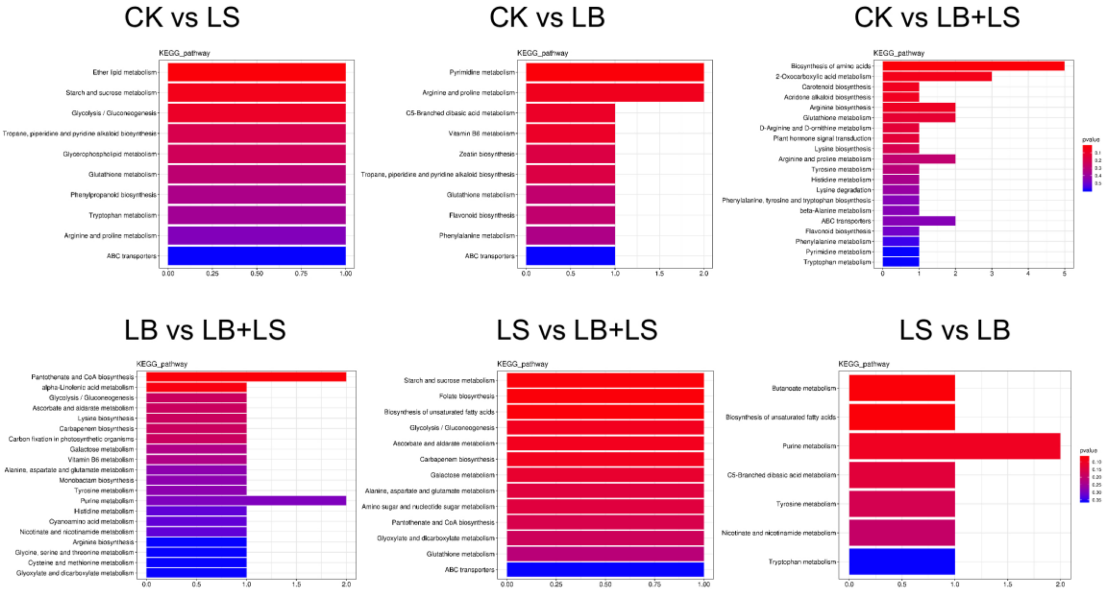

Figure S3. KEGG pathway enrichment analysis of *R. palmatum* in each comparison.

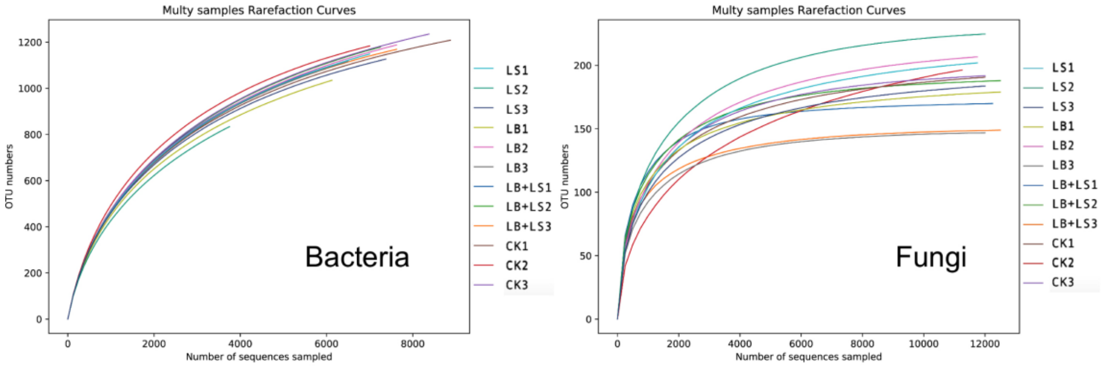

Figure S4 Rarefaction curves of the *R. palmatum* rhizosphere microbial communities.

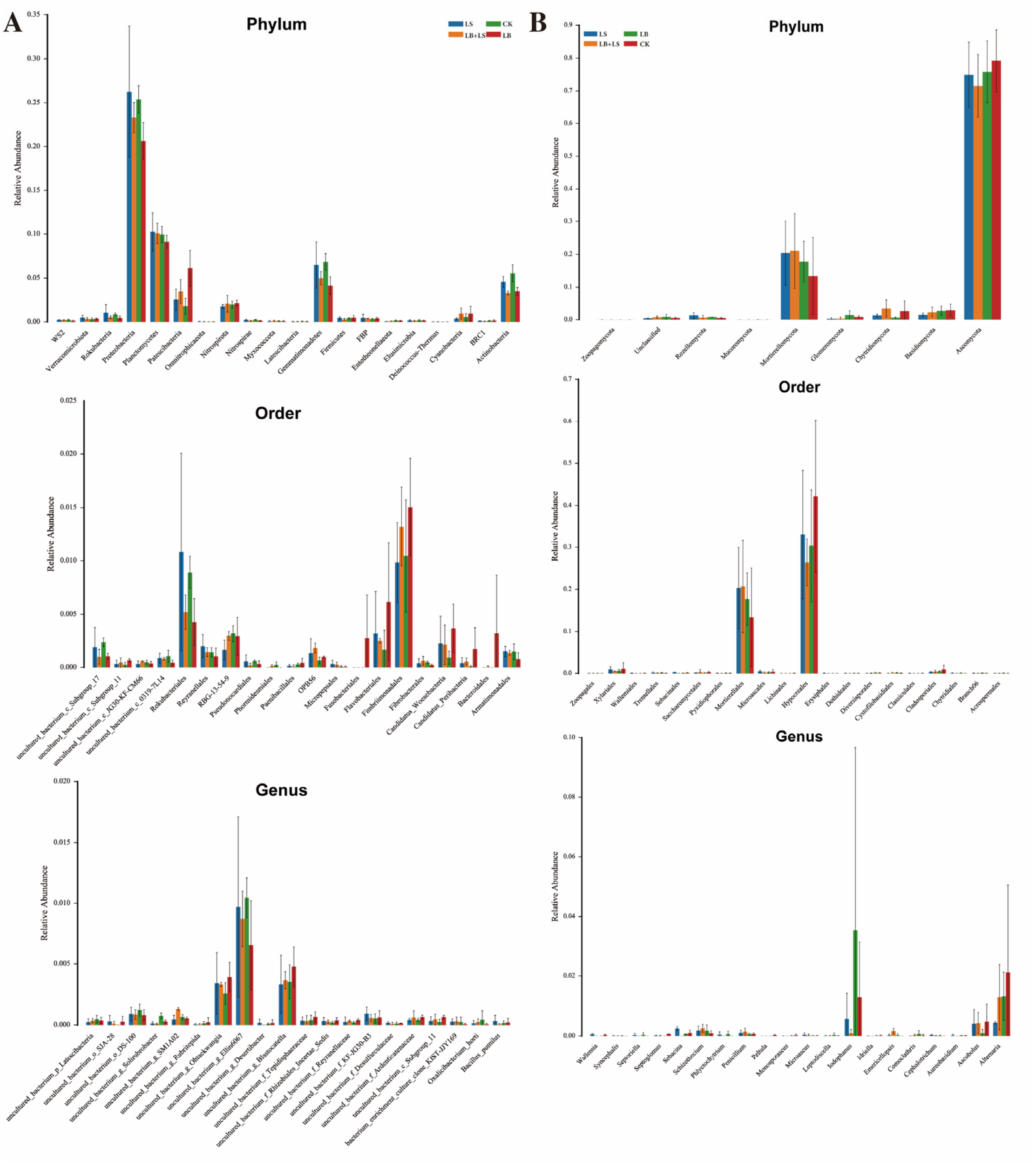

Figure S5 The relative abundances of the top-20 classified microbial phyla, orders, and genera present in the soil samples. Values are the mean ± SE.

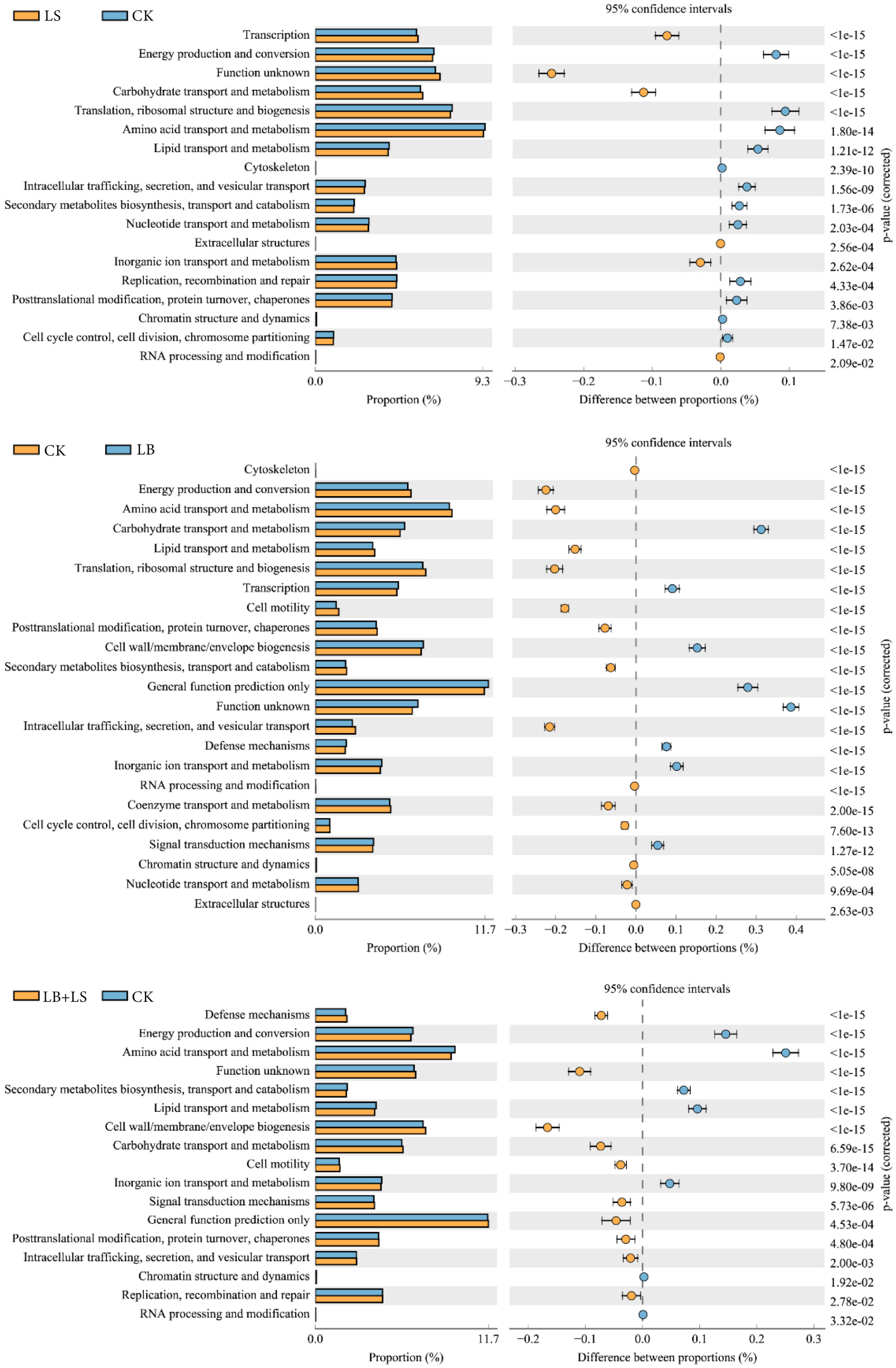

Figure S6 The COG functional classification of bacterial communities as predicted by PICRUSt.

Table S1 Metabolites' classification of the *R. palmatum* metablome.

Table S2 DEMs identified in the widely targeted metabolomics of *R. palmatum* metablome.

Table S3 KEGG pathway classification of the derived *R. palmatum* metablome.

Table S4 Results for the 16S rRNA gene-based bacterial sequencing.
