# Supplementary figures and images for: Bacterial Inoculant and Sucrose Amendments Improve the Growth of Rheum palmatum L. by Reprograming Its Metabolite Composition and Altering Its Soil Microbial Community
Source: Int J Mol Sci. 2022 Feb 1;23(3):1694. doi: 10.3390/ijms23031694 (PMC8835959; doi:10.3390/ijms23031694)

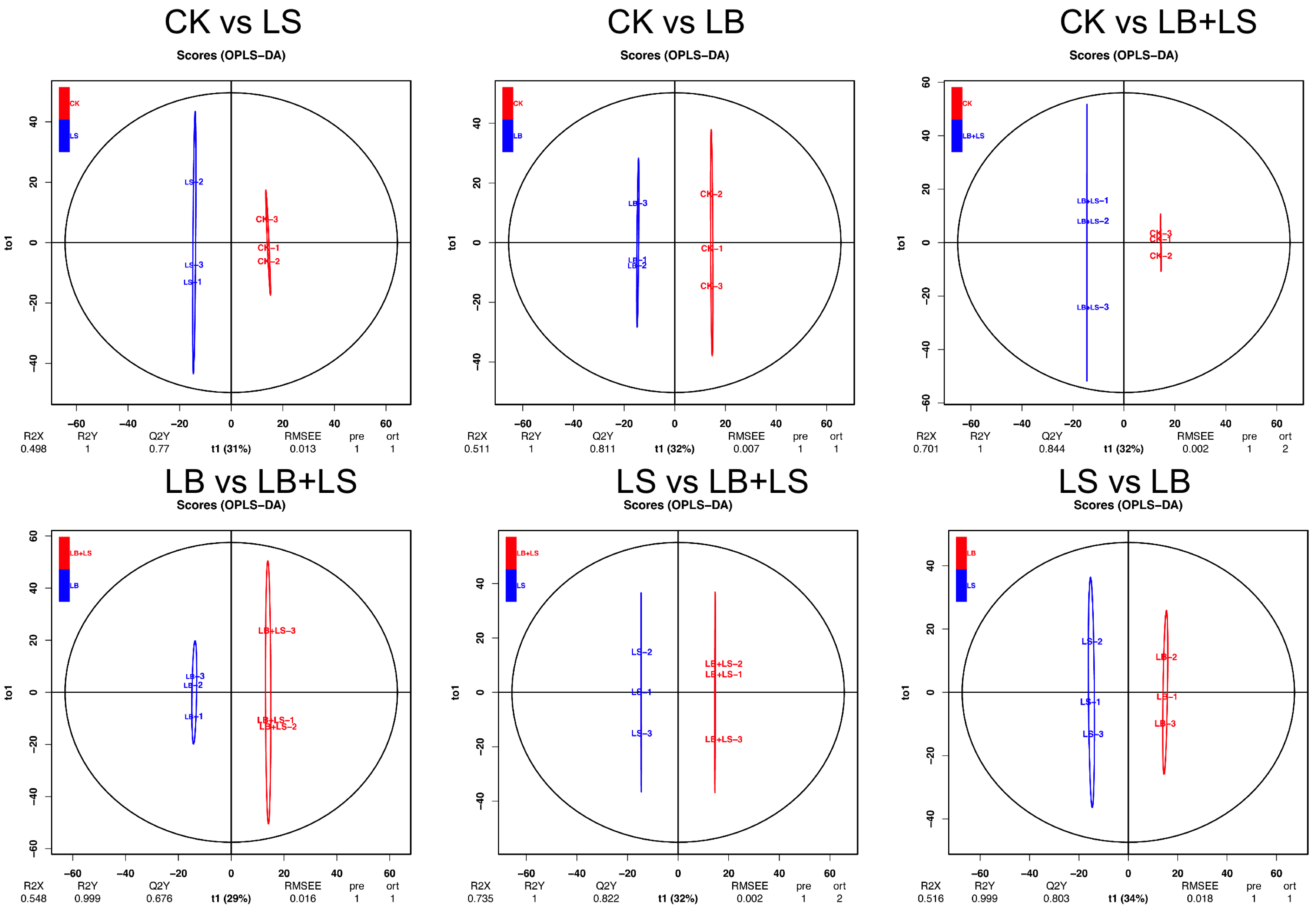

Supplement: Supplementary file 1 [file ijms-23-01694-s001.zip › Supplementary Materials/Supplementary Figures/FigureS1.tif]

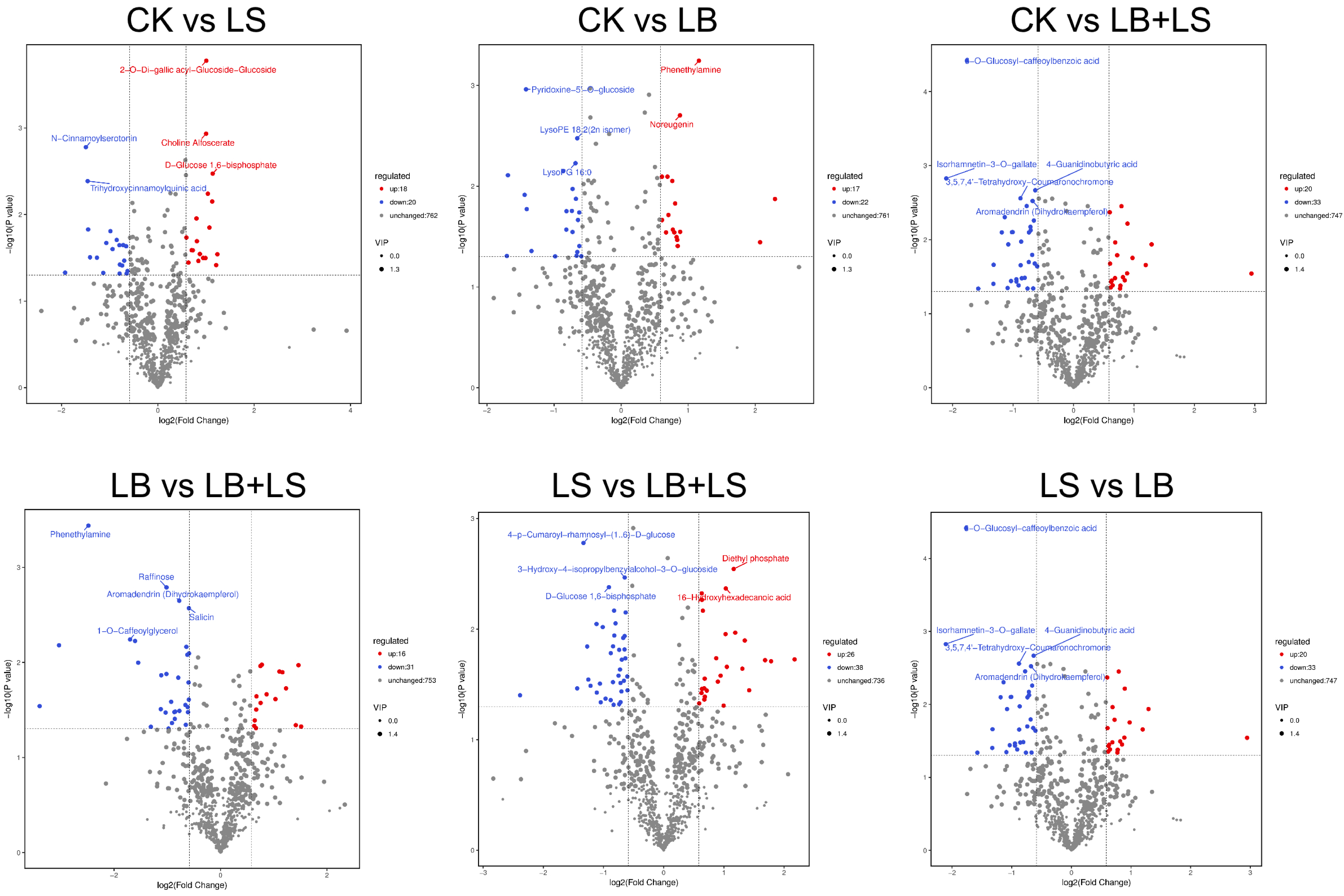

Supplement: Supplementary file 1 [file ijms-23-01694-s001.zip › Supplementary Materials/Supplementary Figures/FigureS2.tif]

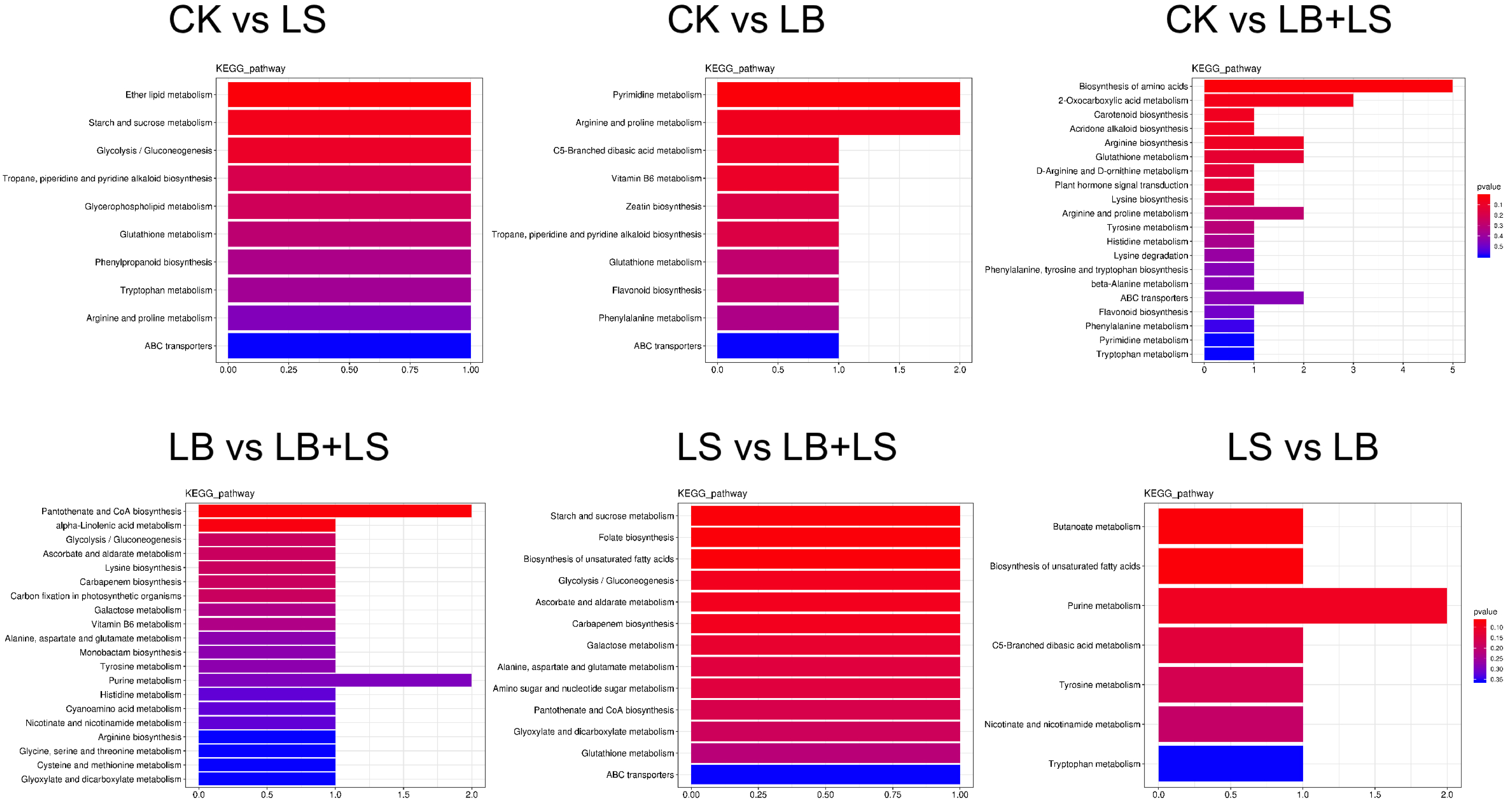

Supplement: Supplementary file 1 [file ijms-23-01694-s001.zip › Supplementary Materials/Supplementary Figures/FigureS3.tif]

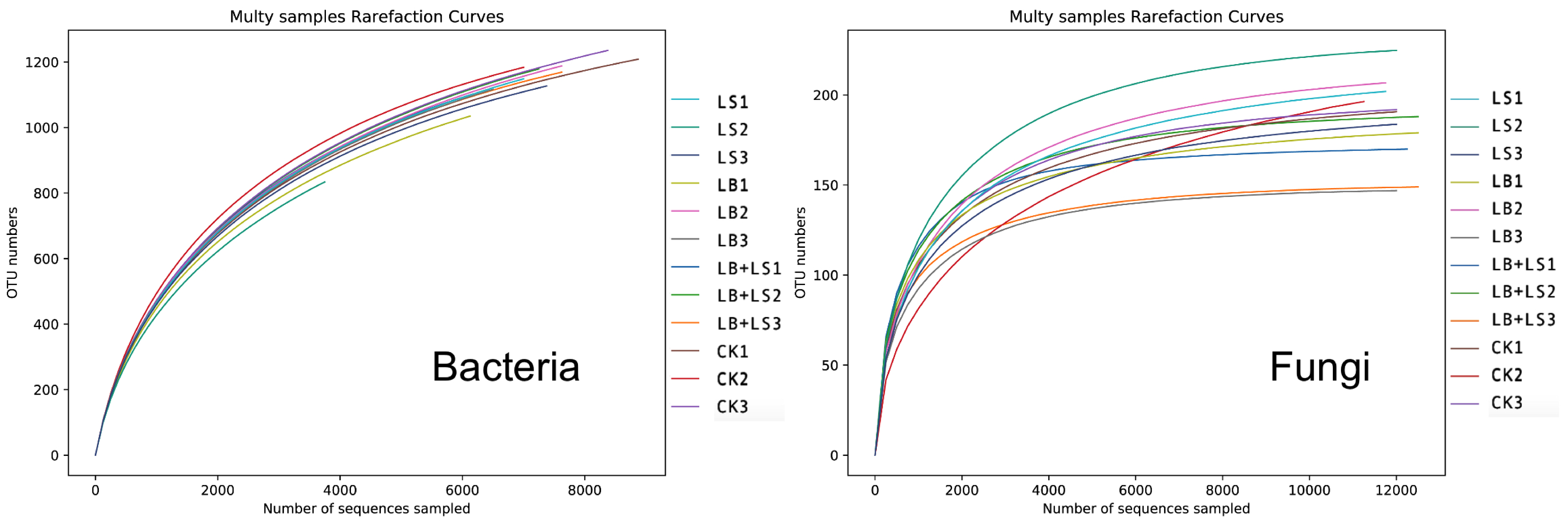

Supplement: Supplementary file 1 [file ijms-23-01694-s001.zip › Supplementary Materials/Supplementary Figures/FigureS4.tif]

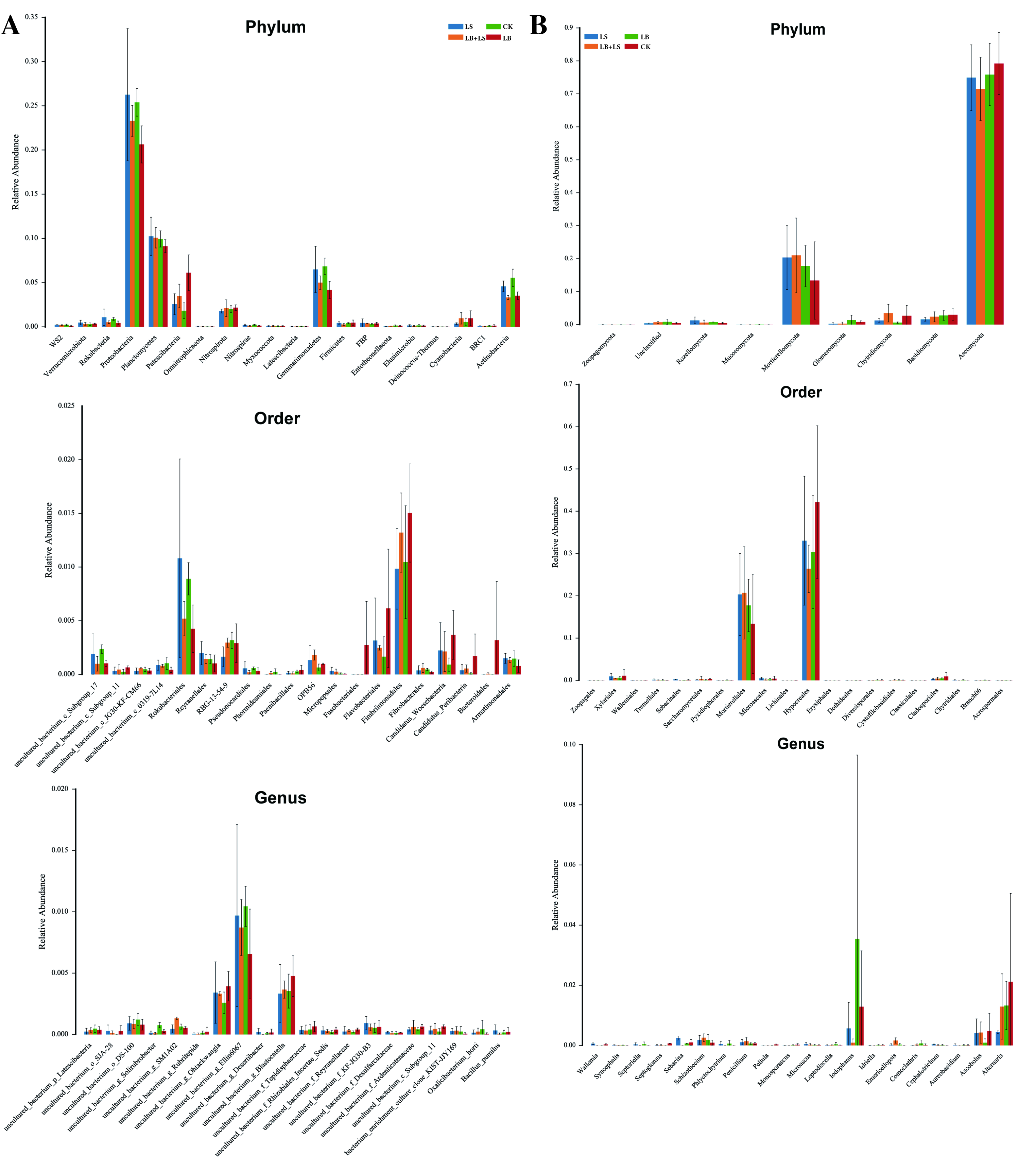

Supplement: Supplementary file 1 [file ijms-23-01694-s001.zip › Supplementary Materials/Supplementary Figures/FigureS5.tif]

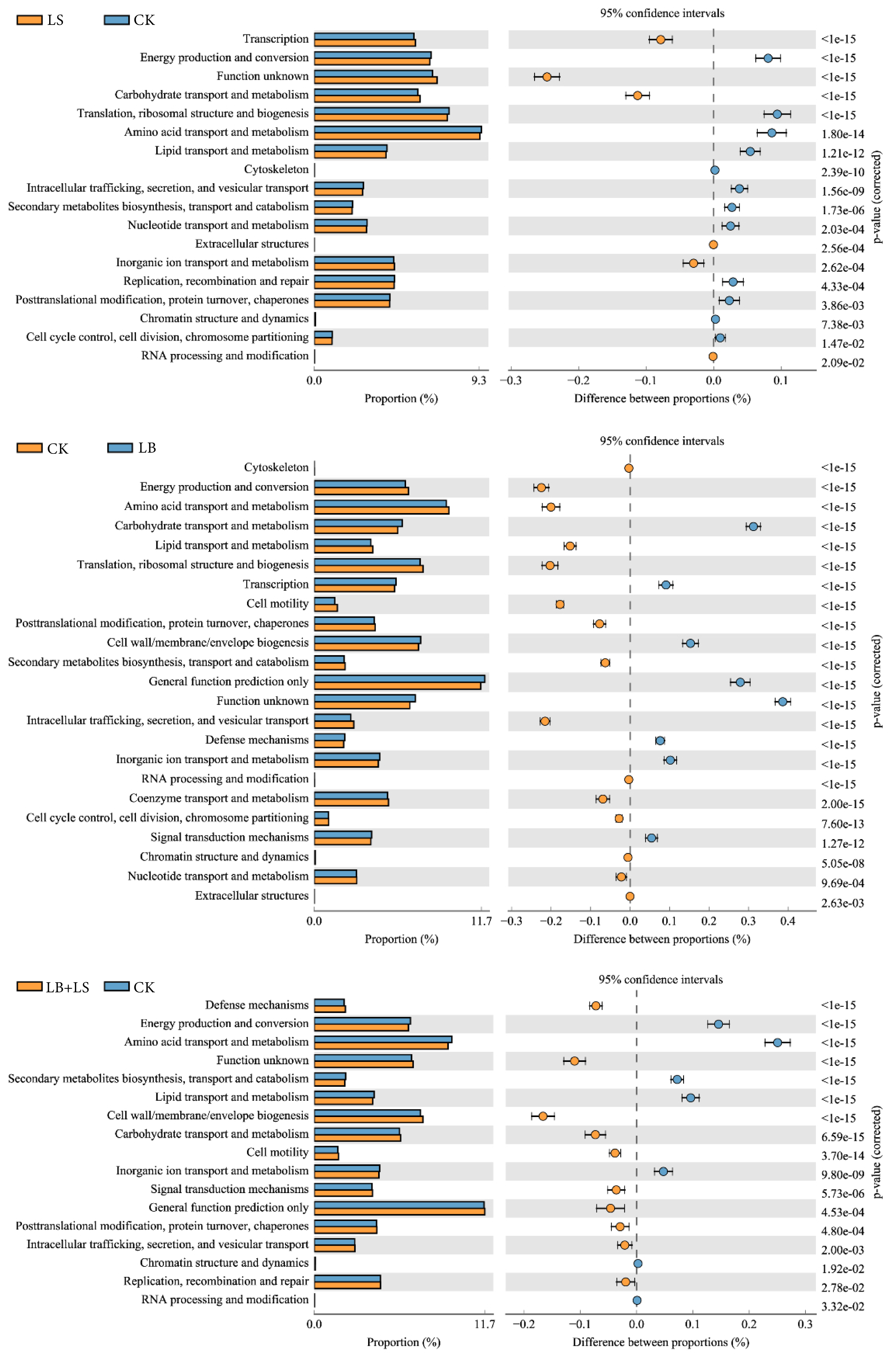

Supplement: Supplementary file 1 [file ijms-23-01694-s001.zip › Supplementary Materials/Supplementary Figures/FigureS6.tif]
